# Screening of Antiviral Components of Ma Huang Tang and Investigation on the Ephedra Alkaloids Efficacy on Influenza Virus Type A

**DOI:** 10.3389/fphar.2019.00961

**Published:** 2019-09-10

**Authors:** Wenyang Wei, Haixia Du, Chongyu Shao, Huifen Zhou, Yiyu Lu, Li Yu, Haitong Wan, Yu He

**Affiliations:** ^1^College of Pharmaceutical Science, Zhejiang Chinese Medical University, Hangzhou, China; ^2^College of Basic, Medical Science, Zhejiang Chinese Medical University, Hangzhou, China; ^3^College of Life Science, Zhejiang Chinese, Medical University, Hangzhou, China; ^4^Institute of Microbiology, Zhejiang Center for Disease Control and Prevention, Hangzhou, China

**Keywords:** Ephedra alkaloids, influenza A virus, virus load, inflammation response, TLRs signal pathway

## Abstract

Although Ma Huang Tang (MHT) has long been considered as a classical formula for respiratory infections like influenza, bronchitis and asthma, its chemical ingredients that really exert the main efficacy are still obscure. In this study we aimed to screen its antiviral components and investigate the potential mechanisms. The MDCK cellular research results showed that, among nine predominant ingredients of MHT, L-methylephedrin (LMEP), L-ephedrine (LEP) and D-pseudo- ephedrine (DPEP) significantly inhibited the proliferation of influenza A virus *in vitro*, and the inhibitory effect at 24 h after the treatment was more obvious than that at 48 h. They also significantly inhibited the mRNA expression levels of related genes in the TLR3, TLR4 and TLR7 signaling pathways, which were accompanied with the down-regulation of TNF-α level and the up-regulation of IFN-β level in the cell supernatant. Therefore, three Ephedra alkaloids exert an antiviral effect *in vitro* which may be closely related to the inhibition of viral replication and the modulation of inflammatory response. Animal research further indicated, at the 3rd and 7th days after infection, LEP and DPEP significantly attenuated lung injury, decreased lung index, virus load in the lung and the level of IL-1β in serum, inhibited the mRNA expression levels of TNF-α, TLR3, TLR4, TLR7, MyD88, NF-κB p65 and RIG-1 as well as the protein expression levels of TLR4, TLR7, MyD88 and NF-κB p65 and markedly increased thymus index, the level of IL-10 in serum and the mRNA expression level of IFN-γ. LEP and DPEP have certain protective effects on the influenza virus-infected mice, which may be associated with their abilities of effectively alleviating lung injury, improving the immunologic function of infected mice and adjusting the host’s TLRs and RIG-1 pathways. The overall findings demonstrate that, as effective and inexpensive natural substances, Ephedra alkaloids and MHT may have potential utility in clinical management.

## Introduction

Influenza, one of the most common human respiratory viral infections, has caused a heavy social and economic burden for centuries (Mehrbod et al., 2014). On a global scale, influenza A virus usually gives rise to severe epidemics of respiratory illness accompanied by sneezing, sore throat, fever, headache, muscle fatigue and inactivity (Ding et al., 2014). Furthermore, the high mutation rate of influenza A virus genes, effective virus transmission, limited efficacy of presently available therapies and rapid emergence of drug resistance may result in more severe symptoms such as respiratory failure, multiple organ dysfunction and even death (Ding et al., 2014). Although vaccination programs are important for preventing and controlling influenza, vaccines lose their efficacy when there are antigenic mismatches with the circulating viruses or when they are administered to high-risk groups, such as the elderly and infants (Jones et al., 2016). Anti-influenza drugs therefore represent a critical and additional line of defense against seasonal influenza viruses and emerging subtypes for which no vaccine may be available. Nowadays, the U.S. Food and Drug Administration licenses four classes of drugs that are effective against influenza A virus, including RNA polymerase inhibitors (ribavirin), M2 channel blockers (amantadine and rimantadine), neuraminidase inhibitors (zanamivir and oseltamivir) and PA inhibitor baloxavir marboxil (Xofluza) (Lee and Yen, 2012; Afzal et al., 2016; FDA NEWS RELEASE, 2018). However, the adverse effects, the limited supply, the compliance problems and the high cost are the major concerns of these drugs. In addition, with the emergence of stable and transmissible drug-resistant strains, such as A/H1N1, drug resistance to these antiviral drugs has emerged and their efficacy may be limited in the future (Ilyushina and Donnelly, 2014; Schnirring, 2019). In view of the limited capability of currently available anti-influenza drugs, it is urgent and time-consuming to develop new antiviral drugs for influenza treatment. Traditional Chinese medicines have unique advantages and broad prospects in terms of cytopathic effects (CPE) caused by viral infection, regulation of immune response, improvement of pulmonary circulation and anti-inflammation (Choi et al., 2017). Therefore, it is of great significance for human health to find an anti-influenza compound, single drug or active ingredient with good therapeutic effects, low toxicity and few side effects from traditional Chinese medicines, and to explore their potential antiviral mechanism.

MHT, a famous classical prescription, has been widely used to treat lung diseases such as influenza, fever, headache, bronchitis, asthma and many other respiratory infections (Ma et al., 2014; Zheng et al., 2015a). Modern clinical research has also proved that MHT has been extensively applied for the treatment of seasonal influenza (Yoshino et al., 2019). In addition to China, MHT has been used in the treatment of influenza and asthma for many years in Korea and Japan (Lim et al., 2016; Kitagawa et al., 2019). Our previous study has demonstrated that MHT could strikingly ameliorate influenza A virus pneumonia in mice, which was associated with the regulating effect of MHT in the imbalance of the body’s immune function and the myeloid differentiation factor 88 (MyD88)-dependent signaling pathway of toll-like receptor (TLR) 4 (Wei et al., 2018a). Furthermore, MHT has also been shown to decrease virus load and relieve CPE in influenza virus-infected MDCK cells by inhibiting the biosynthesis of influenza virus, and to inhibit the mRNA expression of correlative genes in the TLR4 and TLR7 signaling pathways (Wei et al., 2018b).

**Figure 1:**
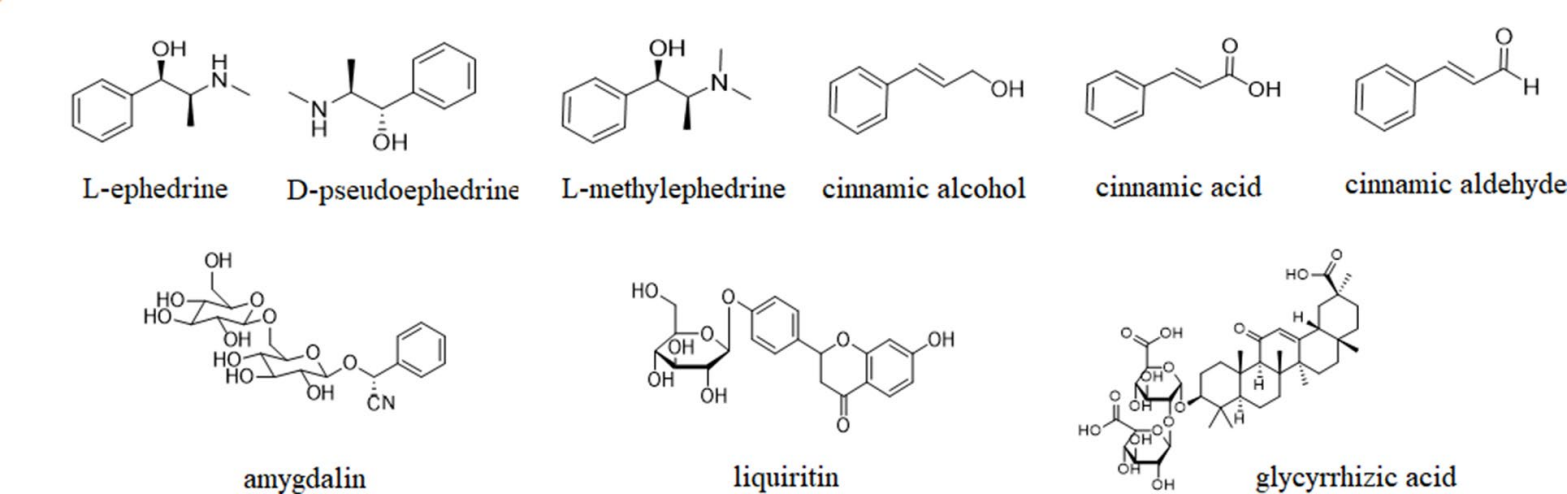
The chemical structures of nine major components in MHT.

Although MHT has long been considered as a typical formula for cold, its chemical ingredients that exert the main efficacy are still obscure. Modern pharmacological studies show that the nine components, including L-ephedrine (LEP), D-pseudoephedrine (DPEP), L-methylephedrin (LMEP), cinnamic alcohol (CAO), cinnamic acid (CMA), cinnamic aldehyde (CMD), amygdalin (AMY), liquiritin (LIQ) and glycyrrhizic acid (GA), are major bioactive ingredients by which MHT produces efficacy, and they are relatively high in content (He et al., 2014; Yu et al., 2017; Wei et al., 2018a) (their chemical structures are shown in **Figure 1**). Therefore, in the present study, the influenza virus-infected MDCK cells were utilized as carriers to evaluate the *in vitro* antiviral effects of nine predominant ingredients, and the potential mechanisms were principally elucidated both *in vitro* and *in vivo*, with the aim to screen the antiviral components of MHT, as well as provide a certain basis for further research and theoretical references for the clinical rational use of MHT.

## Materials and Methods

### Chemicals and Reagents

Oseltamivir (1 caps. = 98.5 mg Oseltamivir Phosphate, Batch no. B3017) was obtained from F. Hoffmann-La Roche Co., Ltd. (Basel, Switzerland). LMEP (99.7%, Batch no. 171247-200301), LEP (99.9%, Batch no. 171241-201007) and DPEP (99.6%, Batch no. 171237-201208) were purchased from the National Institute for the Control of Pharmaceutical and Biological Products (Beijing, China) with the certification of Zhejiang Chinese Medical University. CAO (≥99%, Batch no. ZJ0619BA13), CMA (≥98%, Batch no. Y19A6C2481), CMD (≥99%, Batch no. AJ0620LA13), AMY (≥98%, Batch no. K01020CB14), LIQ (≥98%, Batch no. Z01027BA14), GA (≥98%, Batch no. 20120910), dimethyl sulfoxide solution (DMSO), 3-(4,5-dimethylthiazol- 2-yl)-2,5-diphenyl-tertazoliumbromide (MTT), ELISA kits for canine interleukin (IL)-6 (Batch no. 20170603A), tumor necrosis factor alpha (TNF-α) (Batch no. 20170588A), interferon (IFN)-β (Batch no. 20170502A) and for mouse IL-1β (Batch no. 20171102A), IL-6 (Batch no. 20176020A) and IL-10 (Batch no. 201714899A) were all obtained from Shanghai Yuanye Biotechnology Co., Ltd. (Shanghai, China). The minimum essential medium (MEM), fetal bovine serum (FBS), L-glutamine (Lg), penicillin G (100 IU/ ml) and streptomycin (10000 μg/ml) solution (PS), phosphate-buffered saline (PBS) and 0.25% Trypsin-EDTA solution were purchased from Life Technologies Co., Ltd. (California, USA). High Pure Viral Nucleic Acid Kit was purchased from Roche Co., Ltd. (Mannheim, Germany). SYBR Premix Ex Taq^™^ II (Tli RNaseH Plus) and PrimeScript^™^ RT Master Mix Kit were obtained from TAKARA Bio Inc. (Kusatsu, Japan). Heparin sodium (Batch no. 20161016) was purchased from China Medicine Group Chemical Reagent Co., Ltd. (Hangzhou, China). Antibodies of TLR4 (130 kDa), TLR7 (140 kDa), MyD88 (33 kDa) and nuclear factor (NF)-κB p65 (65 kDa) were purchased from Cell Signaling Technology (Danvers, Colorado, USA), and β-actin (45 kDa) antibody was obtained from Santa Cruz Biotech Co., Ltd (California, USA). The HRP-conjugated Goat Anti-Rabbit/mouse IgG was obtained from Beijing Kangwei Century Biotech Co., Ltd (Beijing, China).

### Virus and Cell

Mouse-adapted influenza virus A/PR8/34 (H1N1) was donated by Professor Yiyu Lu from the Zhejiang Center for Disease Control and Prevention, China. The virus stock was kept at -80°C and its hemagglutination titer was determined to be 1:512. A 50% tissue culture infectious dose (TCID_50_) of influenza A virus strain for MDCK cells was calculated to be 10^-5.28^/0.1 ml. In this present study, 10 TCID_50_ was chosen to infect MDCK cells. MDCK cells were cultivated in MEM containing 10% heat-inactivated FBS, 1% 2 mM Lg and 1% PS in a humidified atmosphere containing 5% CO_2_ at 37 °C.

### Mice

Male ICR mice (18∼22 g) were obtained from the Experimental Animal Center of Zhejiang Chinese Medical University (Hangzhou, China), housed in negative-pressure, HEPA-filtered isolation cabinets with controlled temperature (20 ± 2 °C) and 12 h light/dark cycle. Meanwhile, during the experimental trial, care and experimentation of mice were performed in accordance with the Guide for the Care and Use of Laboratory Animals (NIH Publications, No.80-23, revised in 1996) and the related regulations of Animal Ethics Committee of Zhejiang Chinese Medical University [SYXK (Zhe) 2013-0184].

### Evaluation of Virus Infectivity Titer

A/PR8/34 (H1N1) virus infectivity titer in MDCK cells was measured before further studies. Briefly, confluent MDCK cells were incubated at 37°C with 10-fold serial dilutions of virus, 10 replicates in parallel were designed in each concentration (100 μl/well). The culture medium without FBS was added into the wells (100 μl/well) as the control. Morphological changes were observed under inverted microscope each day, and the number of wells displaying characteristic CPEs was recorded. The wells which displayed a 50% cytopathic rate or more were recorded as CPE wells, while the remainders of the same concentration gradient were recorded as non-CPE wells. After 72 h, CPE wells were counted and virus infectivity titer was calculated as 50% tissue culture infectious dose (TCID_50_) according to the Reed- Muench formula (Su et al., 2016; Wei et al., 2018b).

### Cytotoxicity Assay

MDCK cells with good morphology were seeded in 96-well plates (5 × 10^4^ cells/well) and then treated with LMEP, LEP, DPEP, CAO, CMD, CMA, AMY, LIQ and GA solutions of different concentrations (1.95∼1000 μg/ml), or oseltamivir diluted in FBS-free MEM with different concentrations (0.78∼400 μg/ml). At the same time, 100 μl of FBS-free MEM was taken as the uninfected control. Each group should be designed with 6 replicates. When MDCK cells were cultivated for 48 h at 37 °C in a 5% CO_2_ incubator, 20 μl of 5 mg/ml MTT solution was added into each well and rinsed three times with PBS. After 4 h of incubation, the yellow supernatant in each well was removed and the precipitated formazan crystals were dissolved with 150 μl of DMSO. At 490 nm, the absorbance (A) value of each well was measured on a microplate reader (BIO-RAD, California, USA). The maximal dose of the tested sample with a survival rate higher than 90% was used as the maximal non-toxic concentration (TC_0_). The cell viability rate and the half-maximal toxic concentration (TC_50_) were calculated using the formula described previously (Wang et al., 2013).

### Screening of Effective Components Against Influenza A Virus in MHT

The solutions of nine major components in MHT, from their respective TC_0_, were diluted into medicated solutions of six concentrations with MEM not containing FBS, and 10.00 μg/ml oseltamivir was obtained the same way. All prepared solutions were stored at -20 °C for later use. MDCK cells were cultivated to grow into a single layer in 96-well plates and then inoculated with 10 TCID_50_ of influenza A virus (100 μl/well). After 1 h of incubation at 37°C and 5% CO_2_ atmosphere, the 96-well plates were washed three times with PBS and then the above-mentioned drug solutions with gradient concentrations (100 μl/well). Meanwhile, the control infected (with no drug) and the control uninfected groups (with no virus) were designed, and each experiment group set up six repeat wells. After 48 hours of continuous cultivation, CPE was observed under the inverted microscope. A value was determined according to the steps of the MTT method in the cytotoxicity assay, and cell viability rate was calculated.

### Antiviral Activity Assay *In Vitro*

To evaluate the antiviral activity of LMEP, LEP and DPEP *in vitro*, these three compounds were diluted by MEM not containing FBS into drug solutions at 6 concentrations and stored at -20 °C for later use. MDCK cells were conventionally cultivated in 96 well plates. Then, the antiviral activity assay *in vitro* was carried out using the following four different ways of drug delivery: ① Pre-treatment host cells prior to virus infection: LMEP, LEP, DPEP with 6 concentrations and oseltamivir (10 μg/ml) were added into MDCK cells (100 μl/well). After 1 h of incubation, the overlays were removed. Then, the cell monolayers were washed 3 times with PBS and incubated with 10TCID_50_ influenza A virus (100 μl/well) at 37 °C for 1 h. The virus suspension was removed and replaced by FBS-free MEM after washing 3 times with PBS. ② Limited treatment to 1 hour during virus infection: 50 μl of twofold serially diluted LMEP, LEP, DPEP samples and oseltamivir (10 μg/ml) were added, along with 50 μl of 20 TCID_50_ influenza virus, into the MDCK cell wells, incubated at 37 °C for 1 h, then replaced with MEM containing 1% PS and 1% 2 mM Lg. Subsequently, the 96-well plates processed with the above procedures were incubated at 37 °C in a 5% CO_2_ incubator. ③ Pre-treatment of virus with drug: the two-fold serial dilutions of LMEP, LEP, DPEP and the same amount of 20 TCID_50_ virus suspension were mixed together and incubated at 37 °C in a 5% CO_2_ incubator for 1 h. When MDCK cells grew into confluent monolayer in 96-well plates, the culture medium was removed and the above mixtures were added into the cell wells (100 μl/well). Similarly, the mixture of oseltamivir dilution (10 μg/ml) and the same amount of 20 TCID_50_ virus suspension was added into the positive control wells (100 μl/well). ④ Post-treatment host cells after virus infection: MDCK cells were inoculated with 10TCID_50_ H1N1 influenza virus (100 μl/well) and incubated for 1 h at 37°C and 5% CO_2_ atmosphere. After removing the virus supernatant fluid, each well was washed three times with PBS and overlaid with six concentrations of LMEP, LEP, DPEP and 10 μg/ml oseltamivir (100 μl/well). After 48 h of culture, the CPE induced by H1N1 influenza virus was observed under light microscopy and the antiviral activities of LMEP, LEP or DPEP were measured by MTT reduction assay as described in the cytotoxicity test. For each assay, the control infected and the control uninfected groups were designed, and the mean of six independent measurements for each sample concentration was used for the calculation. The same experiment was repeated three times. The antiviral effective rate (ER), the median efficacious concentration (EC_50_) and the therapeutic index (TI = TC_50/_EC_50_) of LMEP, LEP and DPEP were calculated as described previously (Wei et al., 2018a). The control uninfected group was set at 100%, and the antiviral effective rate of the experimental groups was calculated according to the following equation: antiviral effective rate (ER%) = (mean of A value of experimental group – mean of A value of control infected group)/(mean of A value of control uninfected group –mean of A value of control infected group) × 100%.

### Viral Load Assay Using Real-Time RT-PCR

To quantify the antiviral activity of LMEP, LEP and DPEP, MDCK cells were infected with influenza A virus and simultaneously treated with or without each compound. The detailed procedure for each treatment was as follows. MDCK cells were plated in 24-well plates (2.5×10^5^) and then inoculated with 10TCID_50_ influenza A virus (1 ml/well). After 1 h incubation, the virus supernatant fluid was removed and washed 3 times with PBS. In addition to the control uninfected group, MDCK cells were divided into the control infected group, LMEP-treated groups, LEP-treated groups, DPEP-treated groups and oseltamivir group. Subsequently, the dilutions of LEP, DPEP (15.63, 7.81, 3.91 μg/ml), LMEP (31.25, 15.63, 7.81 μg/ml) and oseltamivir (10 μg/ml) were added into the corresponding cell wells, while the 10TCID_50_ virus suspension and the MEM medium without FBS were added into the control infected and uninfected wells (100 μl/well), respectively. After 24 and 48 h incubation, total viral RNA was isolated from MDCK cells using the High Pure Viral Nucleic Acid kit, and cDNA was synthesized using the PrimeScript^TM^ RT Master Mix kit. Real-time RT-PCR was performed on cDNA samples using the SYBR Premix Ex Tap^™^ II (Takara Bio). The primers of M1 gene targeted on the mRNA of A/PR8/34 influenza virus were synthesized by Sangon Biotech, Co., Ltd. (Shanghai, China) and described in **Table S1**. PCR product level was monitored by 12 K Flex Real-time PCR System (Applied Biosystems; Thermo Fisher Scientific, Waltham, MA, USA), utilizing a amplification protocol consisting of 40 cycles at 95 °C for 2 min, 95 °C for 15 s, 55 °C for 35 s, 55 °C for 15 s and 95 °C for 15 s. Ct value of M1 gene was normalized to the corresponding GAPDH for each sample, and its expression level was calculated using 2^(-ΔΔCt)^ formula (Livak and Schmittgen, 2001), where ΔΔCt = (Ct_target gene_ – Ct_GAPDH_)_experiment_ – (Ct_target gene_ – Ct_GAPDH_)_control uninfected_.

### ELISA Assay of Cytokines in the Cell Supernatant

The supernatant fluid was collected from cultured MDCK cells and treated as described in viral load assay using real-time RT-PCR. Then the IL-6, IFN-β and TNF-α levels in the supernatant solution were measured by ELISA (enzyme-linked immunosorbent assay). The absorbance was measured at 450 nm using an M680 microplate reader (Bio-Rad, USA).

### RT-PCR Detection of the TLRs Pathway

According to our previous studies, the following genes in Toll-like receptor (TLR) pathway were chosen to detection: TLR3, TLR4, TLR7, MyD88, TNF receptor associated factor (TRAF) 3, TRAF6, the interferon regulatory factor 3 (IRF3), NF-κB p65 and IFN-β. For the extraction of total RNA of these target genes, the cDNA synthesis and the concrete RT-PCR operation steps were the same as described for the viral load assay. The primers of the above target genes for canine were designed by Primer 5.0 software, validated by Primer-BLAST in NCBI and synthesized by Sangon Biotech, Co., Ltd. (Shanghai, China) and are described in **Table S1**.

### Preparation of Animal Model and Drug Administration

A total of 108 ICR mice were randomly divided into the control uninfected group, the control infected group, oseltamivir group, LEP-treated groups and DPEP-treated groups; 12 mice were designed in each group. Except for the mice in the control uninfected group, others were anaesthetized with ethyl ether and intranasally infected with 10LD_50_ influenza A virus (A PR/8/34) in a volume of 50 μl PBS to induce viral pneumonia. After 2 h of infection, the mice in the experimental groups were treated by gavage with oseltamivir (22 mg/kg), LEP or DPEP (40, 20, 10 mg/kg) solubilized in physiological saline for 7 days. Meanwhile, the control uninfected group and the control infected group were only given saline with the same volume. The drug dose setting was based on the LEP and DPEP daily dose of the adult 60 kg weight, which was 240 mg per day. The adult daily dose was converted into the high dose of mice, which was about 40 mg/kg. Based on the high dose of the drug, the medium and low doses were set.

### Determination of Lung, Spleen and Thymus Indexes

In the 3rd and 7th days after infection, to monitor the histological changes in influenza A virus-infected animals, six mice were sacrificed randomly in each group, and lung, spleen and thymus tissues were obtained and weighed after being washed with saline solution. Lung index and the inhibition ratio of the lung index were calculated as our previous report, while spleen and thymus indexes were calculated based on the following formulas (Ling et al., 2017).

Spleen index = (spleen weight/body weight)×100%;

Thymus index = (thymus weight/body weight)×100%;

### HE Assay

In the 3rd and 7th days after infection, the right lung lobes from each mouse were washed with normal saline and immediately soaked in 10% normal buffered formalin for one week. Then the samples were dehydrated with ethanol and embedded in paraffin. The 4-μm-thick lung sections were stained with HE and the pathological changes of lung tissue were observed under the light microscope.

### ELISA Analysis of Cytokines in Murine Serum

In days 3 and 7 post-infection, blood samples of six mice from each group were collected from retroorbital sinus under mild ethyl ether anesthesia before they were sacrificed, then under 4 °C they were centrifuged at 3000 rpm for 20 min to obtain serum. The levels of IL-1β, IL-6 and IL-10 in murine serum were determined by ELISA kits.

### RT-PCR Analysis of Viral Load and Related Genes *In Vivo*

In the 3rd and 7th days after infection, the left lung lobes from each mouse were washed with normal saline and stored at -80°C for RT-PCR and western blot analysis use. For RT-PCR analysis, the left lung was homogenized in precooled normal saline and centrifuged at 4000 rpm for 10 min at 4 °C. Total RNA was extracted using the High Pure Viral Nucleic Acid kit and cDNA was synthesized with the PrimeScript^™^ RT Master Mix kit. The specific steps of RT-PCR amplification were the same as the previous section in the present study. The related primers for mouse were designed by Sangon Biotech, Co., Ltd. (Shanghai, China) and are described in **Table S2**.

### Western Blot Assay

The stored left lung tissue was thoroughly cracked with the protein lysis solution containing protease inhibitors, and the total protein was collected after centrifugation to determine the protein concentration. The 40 μg of total protein samples was added in each well and then was transferred to the PVDF membrane after being separated by SDS-PAGE electrophoresis. The PVDF membrane was rinsed 3 times with 0.05% Tween-20 in Tris-buffered saline (TBST) and then blocked for 1 h at room temperature in TBST containing 5% bovine serum albumin. Subsequently, PVDF membrane was incubated overnight with the primary antibody at 4 °C and then incubated with the secondary horseradish peroxidase-linked antibodies after three times of washing. The experiment used β-actin as an internal control and was repeated three times independently. Quantitative analysis of detected bands was performed with Image-J analysis software to observe the expression of the target proteins.

### Statistical Analysis

Results were presented as the mean ± standard deviation (SD) of at least three replicate experiments and all data were analyzed by SPSS 20.0 software. Statistical significance was determined by one-way ANOVA or independent-samples *t*-test for multiple comparisons. Statistical significance was considered at *P* < 0.05.

## Results

### Cytotoxic Effect Assay

It is critical that a novel therapeutic compound has no adverse effect on host-cell growth and cytopathology. Therefore, the cytotoxicity of oseltamivir and nine main bioactive components of MHT for MDCK cells were evaluated by MTT assay. When treated with the tested drugs, the TC_0_ and TC_50_ values of oseltamivir and the nine ingredients in MHT for MDCK cells are shown in **Table S3**. In all of the subsequent experiments, we used the nine components of MHT at TC_0_ or below in FBS-free medium. In addition, 10 μg/ml was chosen as the dose of positive control oseltamivir according to our previous research results (Wei et al., 2018b).

### Effect of the Main Active Ingredients in MHT Against Influenza A Virus *In Vitro*

As shown in **Tables S4, S5 and S6**, after treatment on influenza virus-infected MDCK cells with LMEP, LEP and DPEP, the cell viability rates reached 70% or more at their respective TC_0_. Compared with the control infected group, the changes of the cell viability rates in the CMD and GA were not obvious—only about 47% at their respective TC_0_ (**Tables S7 and S8**). However, CAO, CMA, AMY and LIQ had little antiviral effect on the cell viability of each group after virus infection, as shown in **Tables S9, S10, S11 and S12**.

### Antiviral Effects of LMEP, LEP and DPEP *In Vitro*

**Figure 2:**
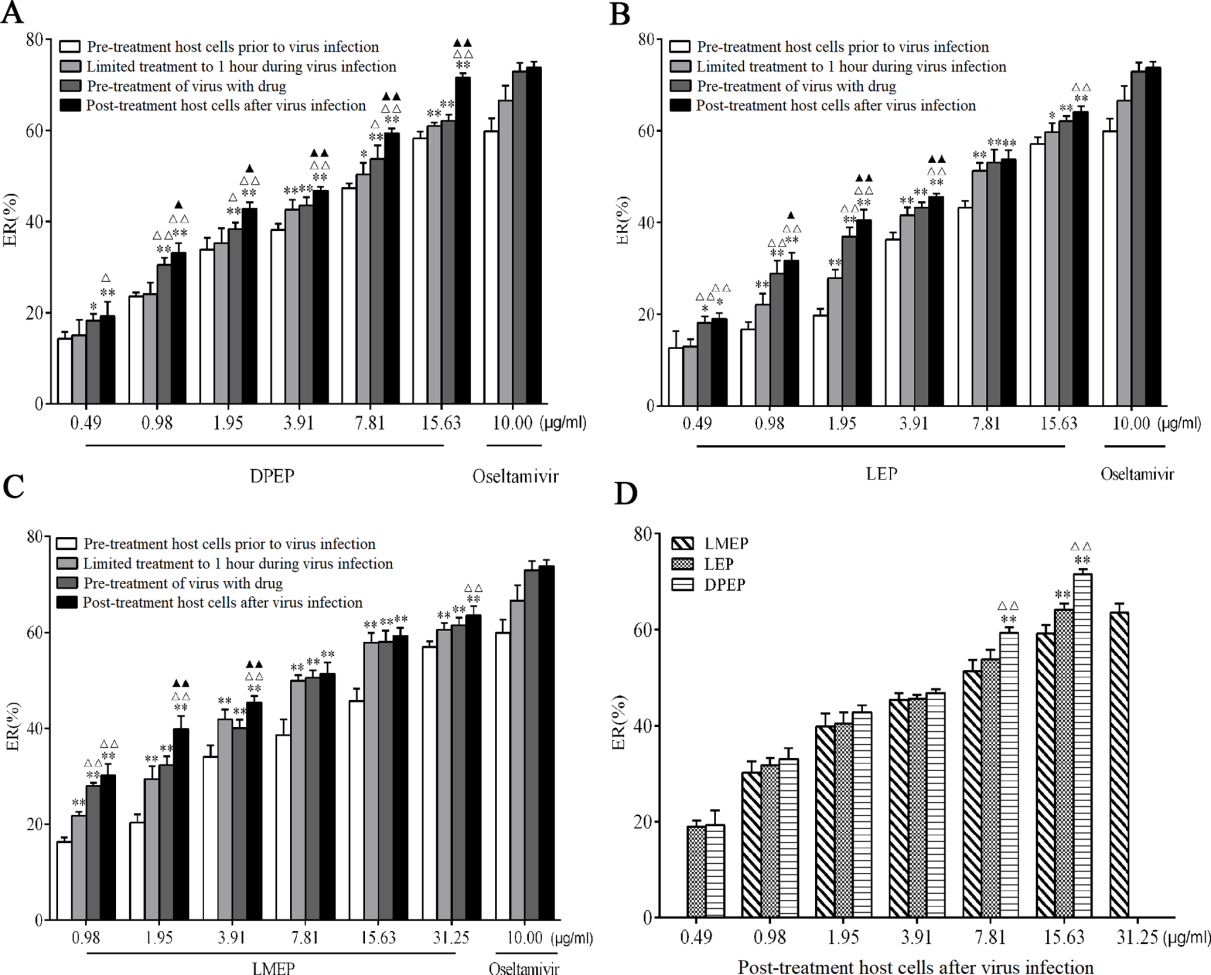
*In vitro* antiviral effects of DPEP, LEP and LMEP against influenza A virus by different ways of drug delivery. MDCK cells (5×10^4^ cells/well) were treated with varying concentrations of DPEP **(A)** and LEP **(B)**, LMEP **(C)** with the ways of pre-treatment host cells prior to virus infection, limited treatment to 1 hour during virus infection, pre-treatment of virus with drug or post-treatment host cells after virus infection. **P < 0.01 compared with the way of pre-treatment host cells prior to virus infection, *P < 0.05 compared with the way of pre-treatment host cells prior to virus infection; ^△△^ *P* < 0.01 compared with the way of limited treatment to 1 hour during virus infection, ^△^ *P* < 0.05 compared with the way of limited treatment to 1 hour during virus infection; ^▲▲^ *P* < 0.01 compared with the way of pre-treatment of virus with drug, ^▲^ *P* < 0.05 compared with the way of pre-treatment of virus with drug. **(D)** ER (%) of DPEP, LEP and LMEP against influenza A virus in the way of post-treatment host cells after virus infection. **P< 0.01 compared with LMEP, *P < 0.05 compared with LMEP; ^△△^ *P* < 0.01 compared with LEP, ^△^ *P* < 0.05 compared with LEP.

Antiviral effects of LMEP, LEP and DPEP on the influenza A/PR8/34 (H1N1) virus were first examined *in vitro*. MDCK cell viability recovered and cytopathological effect in the infected cells was reduced. After LMEP (0.98-31.25 μg/ml) or LEP, DPEP (0.49-15.63 μg/ml) was added to cell cultures in a dose-dependent manner, and influenza virus replication was inhibited. LMEP and LEP, at a final concentration of 3.91 μg/ ml, performed a certain activity against influenza virus with ERs of more than 34.00% and 36.00%, respectively, while ER of more than 38.00% was observed on the same dose of DPEP. The ERs against influenza A virus were 56.94%, 57.10% and 58.30%, respectively, when LMEP (31.25 μg/ml), LEP and DPEP (15.63 μg/ml), at a final concentration, were added to cell cultures by way of pre-treatment host cells prior to virus infection, and the ERs against influenza A virus were 63.52%, 64.16% and 71.57%, respectively, by way of post-treatment host cells after virus infection. Nevertheless, when LMEP, LEP and DPEP were added to cell cultures by way of limited treatment of 1 hour during virus infection, the ERs against influenza A virus reduced to 60.58%, 59.67% and 60.98%. If LMEP, LEP and DPEP were added into cell wells by way of pre-treatment of virus with drug, the ERs against influenza A virus were 61.48%, 62.09% and 62.11% (**Figures 2A‒C**). A less protective effect on infected cells was observed when LMEP, LEP and DPEP were added by way of pre-treatment host cells prior to virus infection, suggesting that the inhibitory activity of LMEP, LEP and DPEP had little effect on events at the cell surface. The viability of infected cells recovered when LMEP, LEP and DPEP were presented by way of limited treatment of 1 hour during virus infection. When incubated with influenza A virus and then added to cell cultures, LMEP, LEP and DPEP showed greater activity of pre-treatment of virus with drug. Compared with the activity of pre-treatment of virus with drug, there was a more significant inhibitory activity found on LMEP, LEP and DPEP when added by way of post-treatment host cells after virus infection (**Figures 2A‒C**). Furthermore, in the present way, the obvious antiviral activity of DPEP was especially significant at the final concentration of 7.81 μg/ml or 15.63 μg/ml compared with LEP and LMEP (*P* < 0.01), and DPEP exhibited similar antiviral activity to oseltamivir (positive control) at a final concentration of 15.63 μg/ ml (*P* > 0.05) (**Figure 2D**). These data indicate that three Ephedra alkaloids inhibit the virus with different ways of action and that the variation of inhibition depends on the application timing of LMEP, LEP or DPEP. Additionally, the EC_50_ and TI of LMEP, LEP and DPEP against influenza A virus *in vitro* were calculated and are shown in **Table 1**.

**Table 1:** EC_50_ and TI of DPEP, LEP and LMEP against influenza A virus *in vitro*.

Drug delivery way	DPEP		LEP		LMEP	
	EC_50_ (μg/ml)	TI	EC_50_ (μg/ml)	TI	EC_50_ (μg/ml)	TI
Blocking viruses invading host cells	8.17	29.04	10.96	19.64	20.37	10.64
Intervening the adsorption of virus	7.57	31.33	7.15	30.13	7.85	27.61
Directly inhibiting virus	6.04	39.27	6.28	34.26	7.55	28.71
Inhibiting the biosynthesis of virus	4.67	50.81	5.66	38.02	6.65	32.58

### Effects of LMEP, LEP and DPEP on Virus Load *In Vitro*

**Figure 3:**
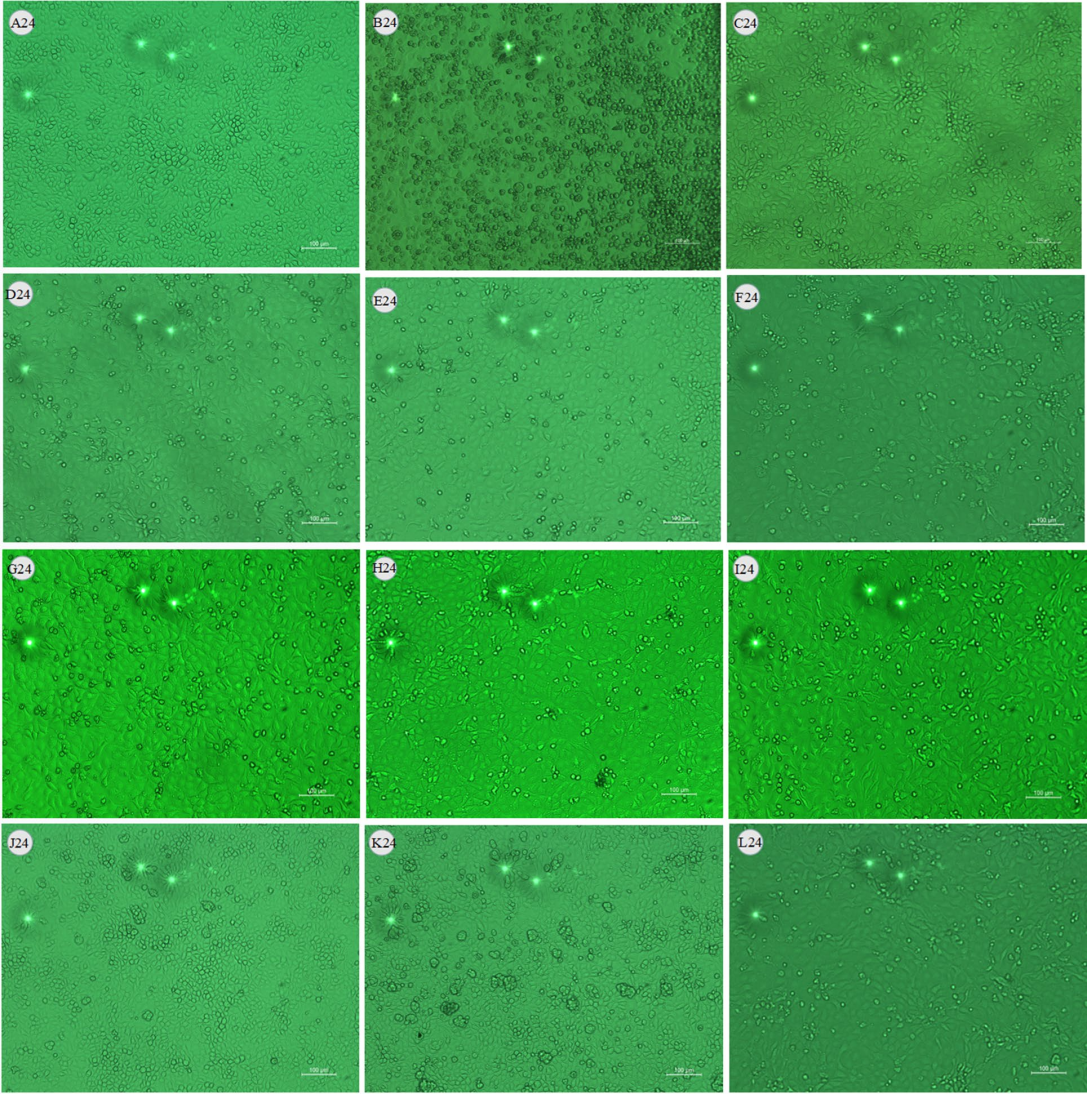
Effects of LMEP, LEP and DPEP on CPE of MDCK cells infected by influenza A virus (×10) after 24 h treatment. **(A)** The control uninfected group; **(B)** the control infected group; **(C)** oseltamivir group; **(D)** LMEP 31.25 μg/ml group; **(E)** LMEP 15.63 μg/ml group; **(F)** LMEP 7.81 μg/ml group; **(G)** LEP 15.63 μg/ml group; **(H)** LEP 7.81 μg/ml group; **(I)** LEP 3.91 μg/ml group; **(J)** DPEP 15.63 μg/ml group; **(K)** DPEP 7.81 μg/ml group; and **(L)** DPEP 3.91 μg/ml group. The number 24 means treatment with LMEP, LEP or DPEP for 24 h.

**Figure 4:**
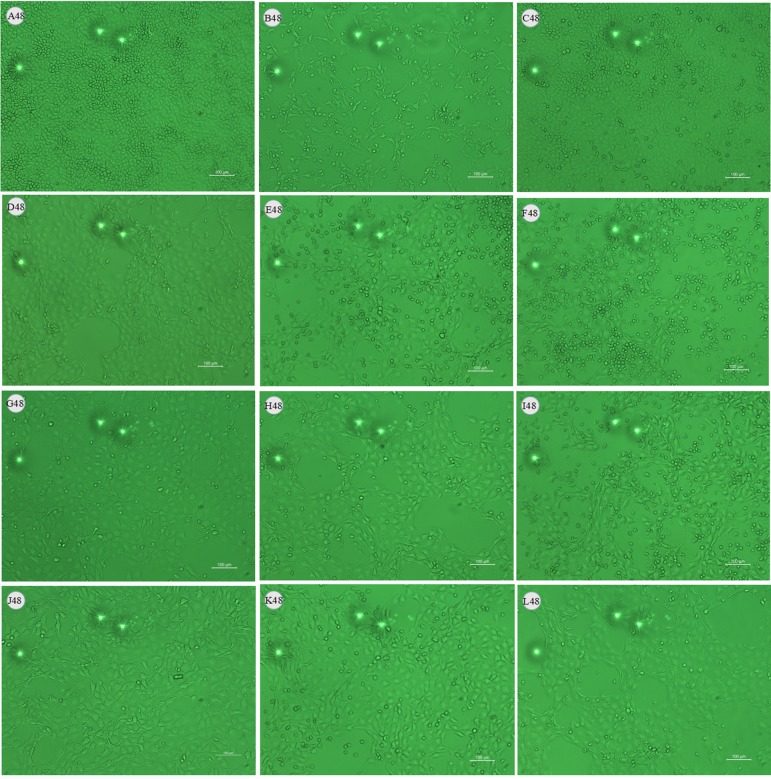
Effects of LMEP, LEP and DPEP on CPE of MDCK cells infected by influenza A virus (×10) after 48 h treatment. **(A)** The control uninfected group; **(B)** the control infected group; **(C)** oseltamivir group; **(D)** LMEP 31.25 μg/ml group; **(E)** LMEP 15.63 μg/ml group; **(F)** LMEP 7.81 μg/ml group; **(G)** LEP 15.63 μg/ml group; **(H)** LEP 7.81 μg/ml group; **(I)** LEP 3.91 μg/ml group; **(J)** DPEP 15.63 μg/ml group; **(K)** DPEP 7.81 μg/ml group; **(L)** DPEP 3.91 μg/ml group. The number 48 means treatment with LMEP, LEP or DPEP for 48 h.

**Figure 5:**
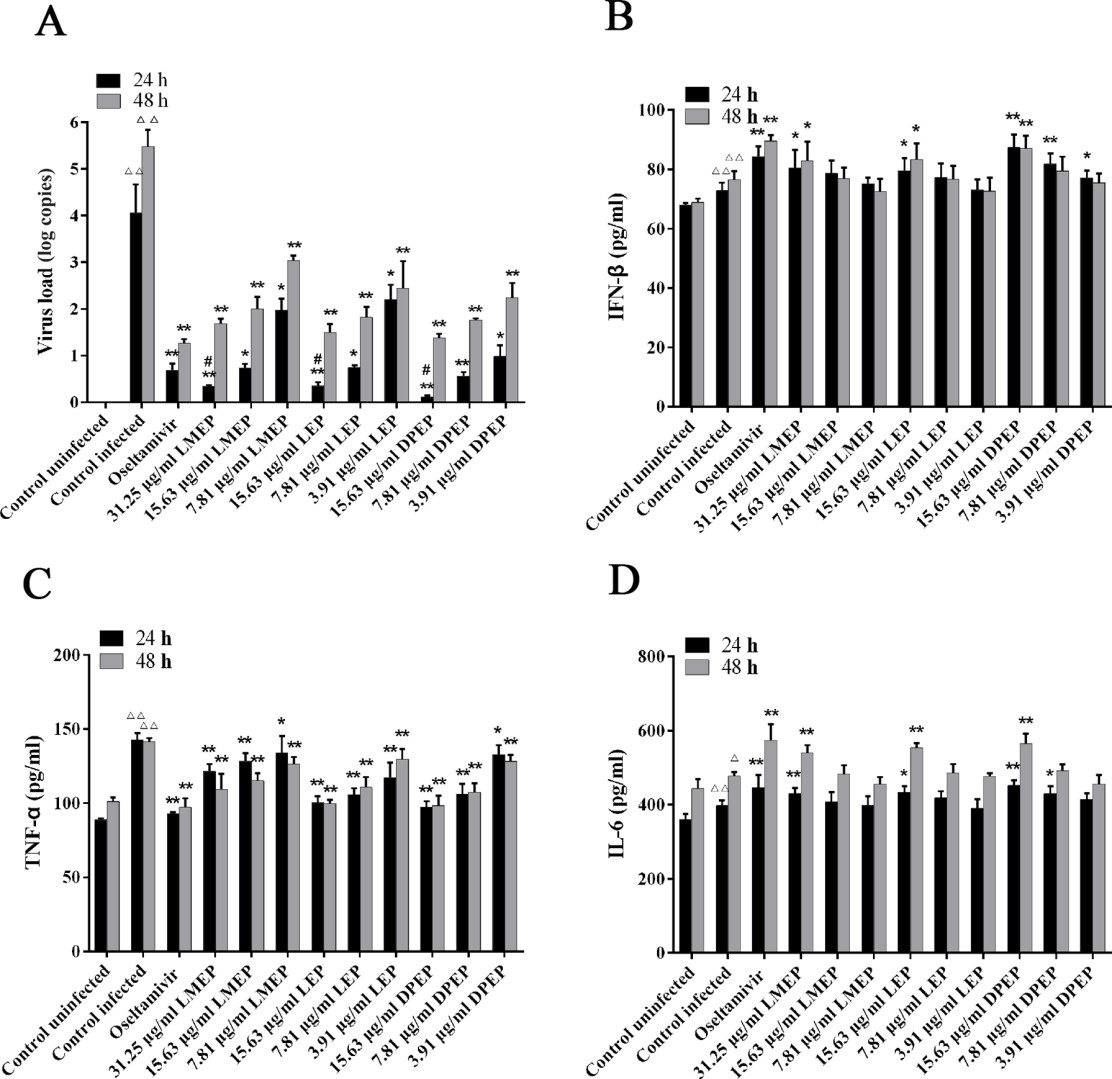
Virus load **(A)** and cytokine expression **(B–D)** in MDCK cells following the treatment of LMEP, LEP and DPEP. ^△△^ *P* < 0.01 compared with the control uninfected group, ^△^ *P* < 0.05 compared with the control uninfected group; **P < 0.01 compared with the control infected group, *P < 0.05 compared with the control infected group; ^#^ *P* < 0.05 compared with oseltamivir group.

After being challenged with influenza A virus, MDCK cells collapsed and detached. Most of them adhered together and suspended on the surface of the culture medium. At 24 h after administration, compared with the control infected group (**Figure 3A** and **Figure 4A**), there were still some MDCK cells remaining in the cell plate (**Figure 3B**), while the CPE at 48 h after administration was more severe than that of 24 h. Influenza A virus infection resulted in the necrosis of almost all the MDCK cells, and adherent cells were depleted (**Figure 4B**). Oseltamivir, LMEP, LEP and DPEP could significantly reduce the CPE and inhibit the influenza virus; additionally, the inhibitory effect for influenza A virus at 24 h after administration was more significant than that at 48 h (**Figures 3C–L** and **Figures 4C–L**). As shown in **Figure 5A**, there was no viral load expression in the cells of the control uninfected group. After H1N1 infection, the viral load in the control infected group was significantly higher than that in the control uninfected group (*P* < 0.01), and that at 48 h after administration was significantly higher than that at 24 h. Compared with the control infected group, the viral load of each dose group of LMEP, LEP and DPEP was significantly decreased at 24 and 48 h after administration (*P* < 0.01 or *P* < 0.05). Moreover, after 24 h treatment, the virus load in the LMEP 31.25 μg/ml, LEP 15.63 μg/ml and DPEP 15.63 μg/ml groups was significantly lower than that of oseltamivir group (*P* < 0.05).

### Effects of LMEP, LEP and DPEP on the Levels of IL-6, TNF-α and IFN-β *In Vitro*

To investigate the effects of LMEP, LEP and DPEP on the regulation of cytokine production, the levels of three inflammatory cytokines were measured from the cell supernatant. After virus infection, the levels of IFN-β, TNF-α and IL-6 were markedly increased compared with the control uninfected group (*P* < 0.01 or *P* < 0.05) (**Figure 5**). When treated with Ephedra alkaloids for 24 and 48 h, the levels of IFN-β and IL-6 were significantly increased, whereas the level of TNF-α was significantly decreased in comparison to the control infected group. Specifically, as shown in **Figure 5B**, 31.25 μg/ml LMEP, 15.63 μg/ml LEP and three doses of DPEP could significantly increase the level of IFN-β after 24 h treatment (*P* < 0.01 or *P* < 0.05), and 15.63 and 7.81 μg/ml DPEP exhibited a similar enhancement effect on the secretion of IFN-β to oseltamivir (all *P* > 0.05). However, except for oseltamivir, the level of IFN-β was only increased by 31.25 μg/ml LMEP, 15.63 μg/ml LEP and 15.63 μg/ml DPEP after 48 h treatment (*P* < 0.01 or *P* < 0.05). Compared with the control infected group, LMEP, LEP and DPEP all significantly inhibited the secretion of TNF-α after 24 or 48 h treatment (*P* < 0.01 or *P* < 0.05). Meanwhile, 15.63 μg/ml LEP and DPEP performed a similar inhibitory effect for TNF-α level to oseltamivir (all *P* > 0.05), as shown in **Figure 5C**. As for IL-6, we observed that, after treatment of 24 and 48 h, its levels in the oseltamivir, LMEP 31.25 μg/ml, LEP 15.63 μg/ml, DPEP 15.63 μg/ml and 7.81 μg/ml groups were significantly higher than that in the control infected group (*P* < 0.01 or *P* < 0.05). Furthermore, there was no significant difference between DPEP 15.63 μg/ml and oseltamivir groups (all *P* > 0.05), as shown in **Figure 5D**.

### Expression of mRNA Related to TLRs Signaling Pathway

**Figure 6:**
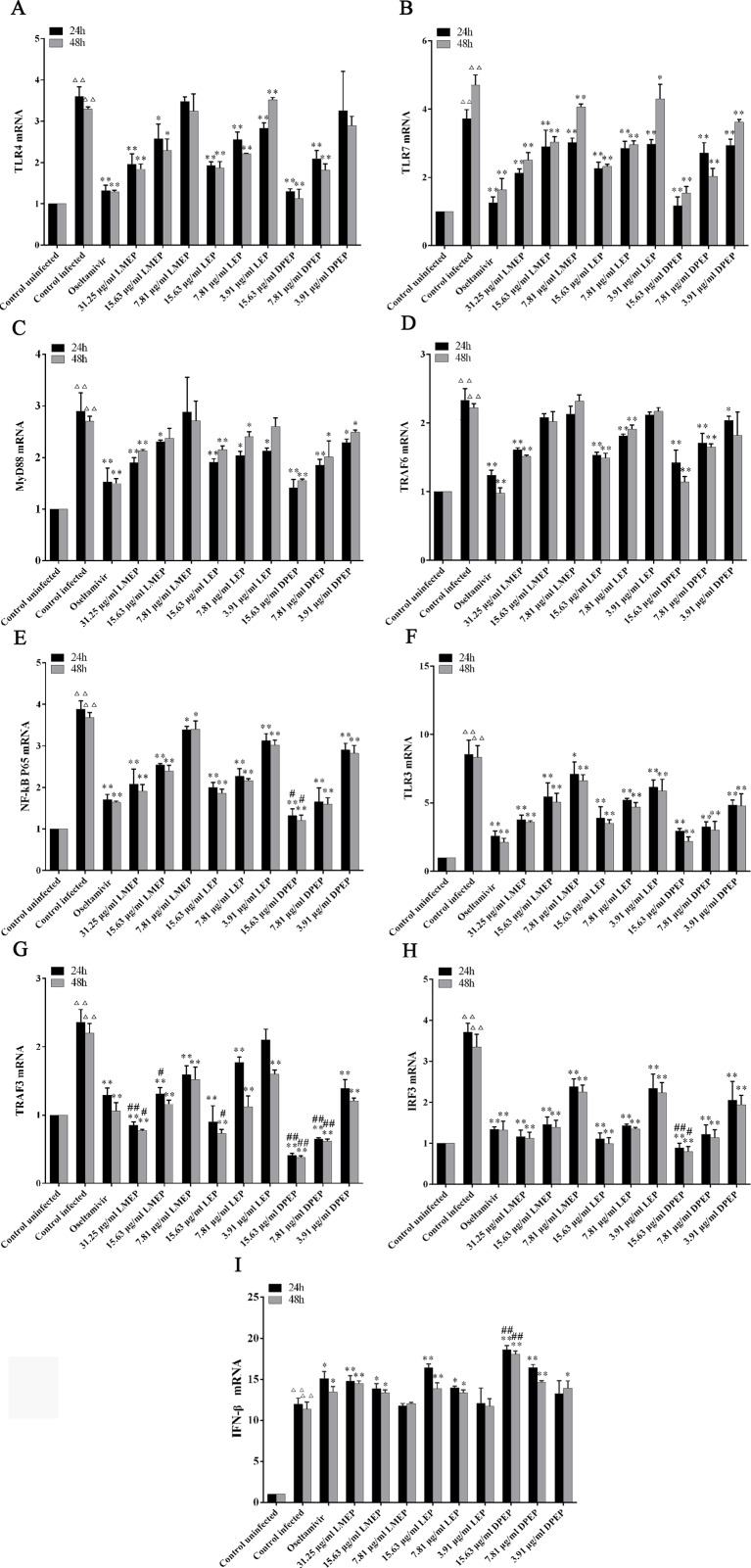
The expression of mRNA related to TLRs signaling pathway. **(A)** TLR4; **(B)** TLR7; **(C)** MyD88; **(D)** TRAF6; **(E)** NF-κB p65; **(F)** TLR3; **(G)** TRAF3; **(H)** IRF3; **(I)** IFN-β. ^△△^ *P* < 0.01 vs. the control uninfected group, **P < 0.01 vs. the control infected group, *P < 0.05 vs. the control infected group, ^##^ *P* < 0.01 vs. oseltamivir group, ^#^ *P* < 0.05 vs. oseltamivir group.

As shown in **Figure 6**, compared with the control uninfected group, mRNA expression levels of TLR3, TLR4, TLR7, Myd88, TRAF6, NF-κB p65, TRAF3, IRF3 and IFN-β in the control infected group were significantly increased (*P* < 0.01). However, mRNA expression levels of TLR3, TLR4, TLR7, Myd88, TRAF6, NF-κB p65, TRAF3 and IRF3 in the drug-treated groups were significantly decreased (*P* < 0.01 or *P* < 0.05), except that the IFN-β mRNA expression was significantly increased (*P* < 0.01 or *P* < 0.05). To be specific, after 24 and 48 h treatment, mRNA expression levels of TLR7, NF-κB p65, TLR3 and IRF3 in three dose groups of LMEP, LEP and DPEP were significantly reduced (*P* < 0.01 or *P* < 0.05), while mRNA expression of IFN-β in the high and medium dose groups of the three alkaloids was significantly increased (*P* < 0.01). After 24 h treatment, obvious decreased mRNA expression levels of MyD88, TRAF6 and TRAF3 were observed in three dose groups of DPEP (*P* < 0.01 or *P* < 0.05), while three doses of LEP significantly inhibited the mRNA expression of TLR4 and MyD88 (*P* < 0.01 or *P* < 0.05). mRNA expression of TRAF6 and TRAF3 was decreased by the 15.63 or 7.81 μg/ml LEP treatment; mRNA expression of TLR4, MyD88 and TRAF3 in the LMEP 31.25 μg/ml and 15.63 μg/ml groups was significantly reduced (*P* < 0.01 or *P* < 0.05); and the significant decreased mRNA expression of TLR4 was also found in the DPEP 15.63 μg/ml and 7.81 μg/ml groups (*P* < 0.01). After 48 h treatment, the significant decreased mRNA expression of MyD88 and TRAF3 was observed in three different dose groups of DPEP (*P* < 0.01 or *P* < 0.05), while mRNA expression levels of TRAF6 and TLR4 were only reduced by 15.63 or 7.81 μg/ml DPEP (*P* < 0.01) treatment. Three doses of LEP only inhibited the mRNA expression levels of TLR4 and TRAF3 (*P* < 0.01), while the marked decreased mRNA expression of MyD88 and TRAF6 were only found in the LEP 15.63 μg/ml and 7.81 μg/ml groups (*P* < 0.01 or *P* < 0.05). Three doses of LMEP all significantly reduced the mRNA expression of TRAF3 (*P* < 0.01), but the significant decreased mRNA expression of TLR4 was only found in the LMEP 31.25 μg/ml or 15.63 μg/ml group (*P* < 0.01 or *P* < 0.05). In addition, compared with the oseltamivir group, mRNA expression levels of NF-κB p65, TRAF3 and IRF3 in the DPEP 15.63 μg/ml group were significantly reduced, while IFN-β mRNA expression was significantly increased after 24 or 48 h treatment (*P* < 0.01 or *P* < 0.05).

### Effects of LEP and DPEP on Lung Index of Mice

At the 3rd day post-infection, mice in the control infected group began to show some infection symptoms, manifesting sluggishness, shortness of breath, arched back, pilomotor fur, contracture, poor appetite, decreased drinking water, and the condition gradually worsened. Compared with the control uninfected group, the lung indexes of mice in the control infected group were significantly increased. However, compared with the control infected group, the lung indexes of mice in the LEP and DPEP groups were significantly reduced (*P* < 0.01 or *P* < 0.05), and the lung index decrease in the 20 mg/kg group of each drug was more obvious than that in the other dose groups (**Table 2**). At the 7th day after infection, the lung index of the control infected group continued to increase significantly compared with the control uninfected (*P* < 0.01), while the lung index of the LEP and DPEP groups decreased to a certain extent (*P* < 0.01 or *P* < 0.05). Furthermore, the marked down-regulation tendency was found in the 40 mg/kg and 20 mg/kg groups of each drug (all *P* < 0.01).

**Table 2:** The inhibitory effect of LEP and DPEP on lung index of infected mice (*n* = *6*).

Groups	Dose (mg/kg^)^	Day 3		Day 7	
		Lung insdex $\left(\bar{x}\;\pm \;s\right)$	Inhibition ratio of lung index (%)	Lung index $\bar{x}\;\pm \;s$	Inhibition ratio of lung index (%)
Control uninfected	/	0.67 ± 0.05	/	0.64 ± 0.04	/
Control infected	/	1.26 ± 0.16^△△^	/	1.93 ± 0.14^△△^	/
Oseltamivir	22	0.74 ± 0.03**	41.27	1.18 ± 0.11**	38.86
LEP-treated	40	0.90 ± 0.05**	28.57	1.45 ± 0.16**	24.87
	20	0.82 ± 0.11**	34.92	1.37 ± 0.06**	29.02
	10	0.97 ± 0.09**	23.02	1.57 ± 0.10**	18.65
DPEP-treated	40	0.89 ± 0.06**	29.37	1.44 ± 0.11**	25.39
	20	0.75 ± 0.13**	40.48	1.39 ± 0.08**	28.00
	10	0.95 ± 0.08**	24.60	1.60 ± 0.23*	17.10

^△△^P < 0.01 compared with the control uninfected group; **P < 0.01 compared with the control infected group, *P < 0.05 compared with the control infected group.

### Effects of LEP and DPEP on Immune Organs in Mice Infected With Influenza A Virus

A notable decrease in the thymus index was observed in the control infected group on both day 3 and day 7 post-infection (all *P* < 0.01), whereas the spleen index decreased vaguely (**Table 3**). When exposed to oseltamivir, LEP or DPEP, the thymus indexes were significantly increased in comparison to the control infected group; thymus indexes in the oseltamivir group as well as the LEP and DPEP 20 mg/kg groups were especially higher than those in the other groups. In addition, there was an obscure tendency for the spleen index to increase after drug administration.

**Table 3:** Effects on spleen index and thymus index of infected mice $\left(\bar{x}\;\pm \;s,\;n=6\right)$

Groups	Dose (mg/kg)	Day 3		Day 7	
		spleen index	thymus index	spleen index	thymus index
Control uninfected	/	0.51 ± 0.02	0.47 ± 0.06	0.45 ± 0.08	0.41 ± 0.05
Control infected	/	0.46 ± 0.07	0.31 ± 0.02^△△^	0.47 ± 0.06	0.27 ± 0.02^△△^
Oseltamivir	22	0.51 ± 0.13	0.45 ± 0.05**	0.49 ± 0.08	0.38 ± 0.03**
LEP-treated	40	0.56 ± 0.04	0.41 ± 0.01*	0.52 ± 0.07	0.34 ± 0.01*
	20	0.53 ± 0.03	0.43 ± 0.02**	0.53 ± 0.02	0.35 ± 0.03**
	10	0.50 ± 0.01	0.36 ± 0.05	0.46 ± 0.02	0.31 ± 0.07
DPEP-treated	40	0.69 ± 0.08	0.41 ± 0.05*	0.51 ± 0.04	0.34 ± 0.05*
	20	0.58 ± 0.01	0.44 ± 0.02**	0.52 ± 0.06	0.37 ± 0.03**
	10	0.54 ± 0.05	0.39 ± 0.07*	0.48 ± 0.07	0.32 ± 0.04*

^△△^P < 0.01 compared with the control uninfected group; **P < 0.01 compared with the control infected group, *P < 0.05 compared with the control infected group; ^##^P < 0.01 compared with oseltamivir group, ^#^P < 0.05 compared with oseltamivir group.

### Effects of LEP and DPEP on Histopathological Responses

**Figure 7:**
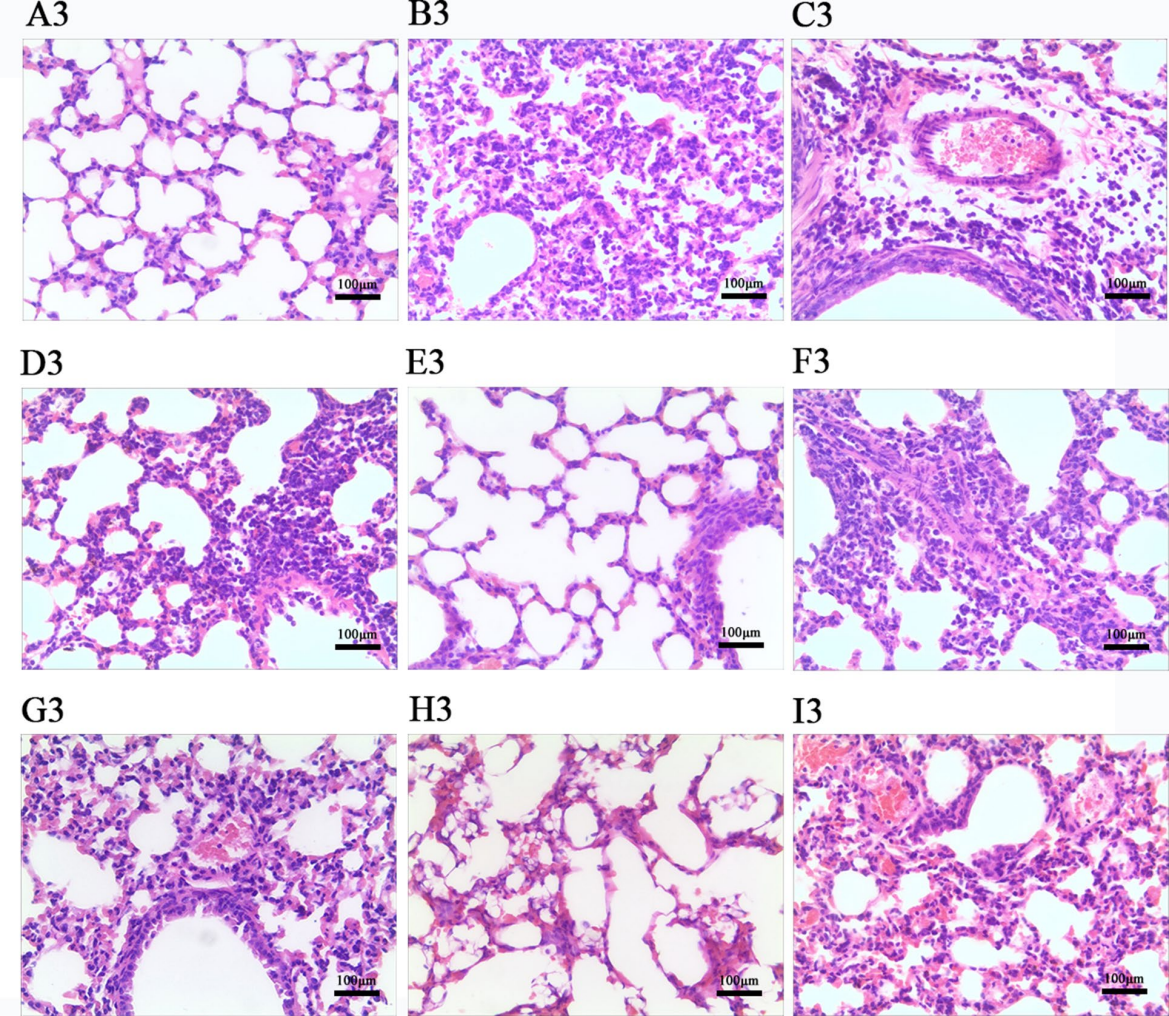
Photomicrographs of lung tissue morphology of mice at day 3 post-infection with HE staining (magnification, 100×). **(A)** The control uninfected group; **(B)** the control infected group; **(C)** oseltamivir group; **(D)** LEP 40 mg/kg group; **(E)** LEP 20 mg/kg group; **(F)** LEP 10 mg/kg group; **(G)** DPEP 40 mg/kg group; **(H)** DPEP 20 mg/kg group; **(I)** DPEP 10 mg/kg group. The number 3 means the 3rd day post-infection.

**Figure 8:**
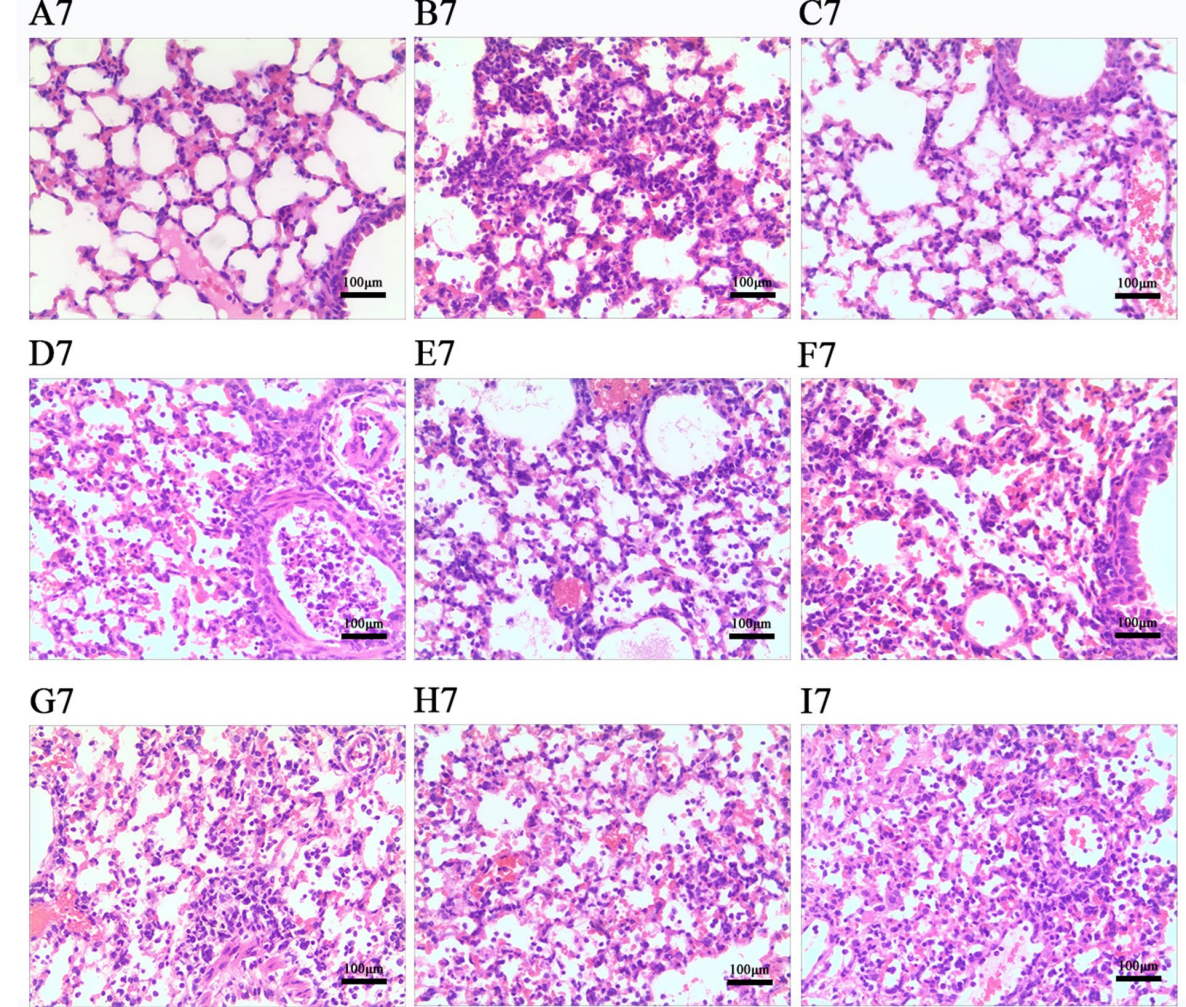
Photomicrographs of lung tissue morphology of mice at day 7 post-infection with HE staining (magnification, 100×). **(A)** The control uninfected group; **(B)** the control infected group; **(C)** oseltamivir group; **(D)** LEP 40 mg/kg group; **(E)** LEP 20 mg/kg group; **(F)** LEP 10 mg/kg group; **(G)** DPEP 40 mg/kg group; **(H)** DPEP 20 mg/kg group; **(I)** DPEP 10 mg/kg group. The number 7 means the 7th day after infection.

According to **Figures 7** and **8**, histopathologic examination of lung tissue showed that the control uninfected mice had normal lungs in terms of size, color and texture. Nevertheless, at days 3 and 7 post-infection, most control infected mice showed severe infiltration of monocytes and lymphocytes in the bronchioles, small vessel walls and surrounding tissues, vasodilation appearing in the interstitial spaces, thickened alveolar walls and exudation of inflammatory cells into the alveolar space. Additionally, more severe pathological damage was observed in murine lung tissue at day 7 post-infection. In contrast, LEP and DPEP treatment attenuated the inflammation, the range and degree of pulmonary damage were significantly reduced, the alveolar walls were slightly thickened inflammatory cell infiltration and congestive bleeding were significantly improved. The alleviating effect at day 3 post-infection was more obvious than that at day 7, and the effects of 20 mg/kg LEP and DPEP were better than 40 or 10 mg/kg LEP and DPEP.

### Effects of LEP and DPEP on Virus Load *In Vivo*

**Figure 9:**
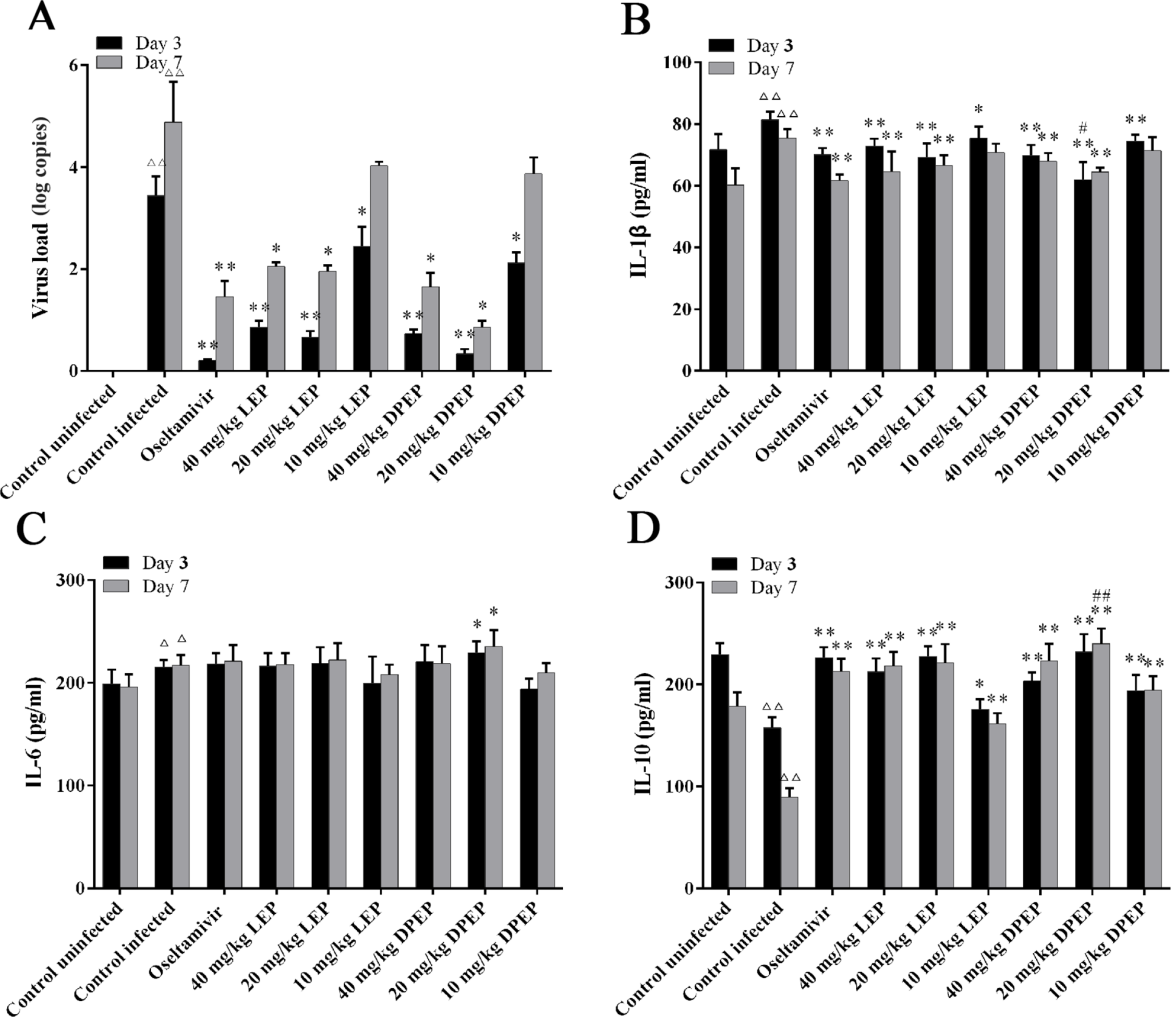
Virus load **(A)** and cytokine expression **(B–D)** in mice following the treatment of LEP and DPEP at days 3 and 7 post-infection. ^△△^ *P* < 0.01 compared with the control uninfected group, ^△^ *P* < 0.05 compared with the control uninfected group; **P < 0.01 compared with the control infected group, *P < 0.05 compared with the control infected group; ^##^ *P* < 0.01 compared with oseltamivir group, ^#^ *P* < 0.05 compared with the oseltamivir group.

As shown in **Figure 9A**, there was no expression of viral load in the lung tissue of the control uninfected mice. At the 3rd and 7th days after infection, the viral load in murine lung tissue of the control infected group was significantly increased (*P* < 0.01), and the virus load at the 7th day was higher than that at the 3rd day. Compared with the control infected group, three doses of LEP and DPEP all significantly decreased the viral load at the 3rd day after infection (*P* < 0.01 or *P* < 0.05). Furthermore, at the 7th day, the viral load in the mice lung tissue from the LEP and DPEP 40 and 20 mg/kg groups was also significantly reduced compared with the control infected group (*P* < 0.05).

### Effects of LEP and DPEP on Cytokine Levels in Serum

Influenza A virus infection is known to induce a strong inflammatory reaction, hallmarked by the production of inflammatory cytokines. Serum was collected at the 3rd and 7th day after infection for the measurement of cytokines. Virus infection caused significantly increased IL-1β and IL-6 as well as significantly decreased IL-10 in the control infected group (*P* < 0.01). After treatment, oseltamivir, LEP and DPEP all dramatically inhibited the production of IL-1β (*P* < 0.01 or *P* < 0.05) and accelerated the production of IL-10 (*P* < 0.01), whereas IL-6 in serum was slightly increased (*P* > 0.05) (**Figures 9B–D**).

### Effects of LEP and DPEP on the mRNA Expression of IFN-γ and TNF-α in Lung Tissues

**Figure 10:**
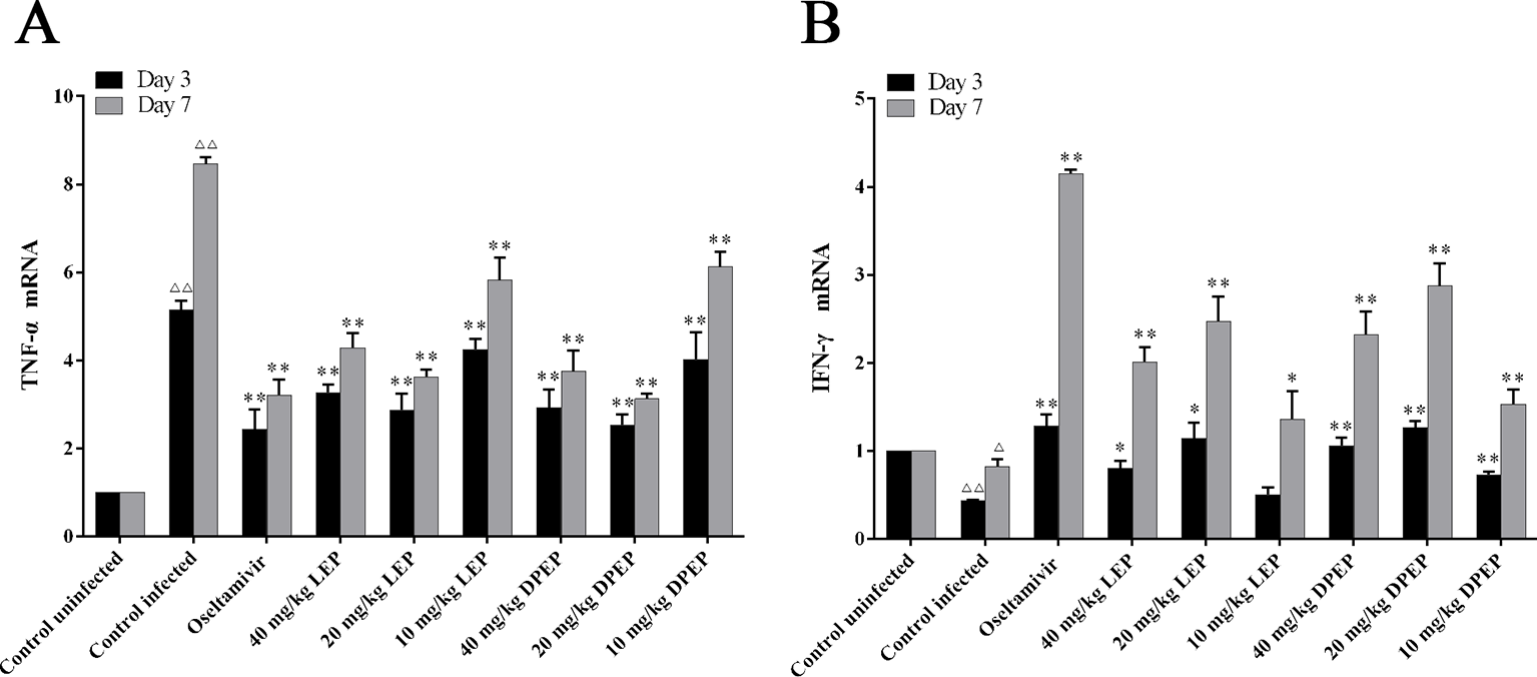
Effects of LEP and DPEP treatment on the mRNA expression of TNF- α **(A)** and IFN-γ **(B)** in lung tissue of mice. ^△△^ *P* < 0.01 compared with the control uninfected group, ^△^ *P* < 0.05 compared with the control uninfected group; **P < 0.01 compared with the control infected group, *P < 0.05 compared with the control infected group.

As shown in **Figure 10**, compared with the control uninfected group, mRNA expression of TNF-α was significantly up-regulated, while mRNA expression of IFN-γ was markedly down-regulated in the control infected group (*P* < 0.01 or *P* < 0.05). In the treatment groups of LEP and DPEP, mRNA expression of TNF-α was markedly inhibited, while mRNA expression of IFN-γ was significantly promoted in comparison to the control infected group (*P* < 0.01 or *P* < 0.05).

### Effects of LEP and DPEP on mRNA Expression of Genes Related to NF-κB Signaling Pathway

**Figure 11:**
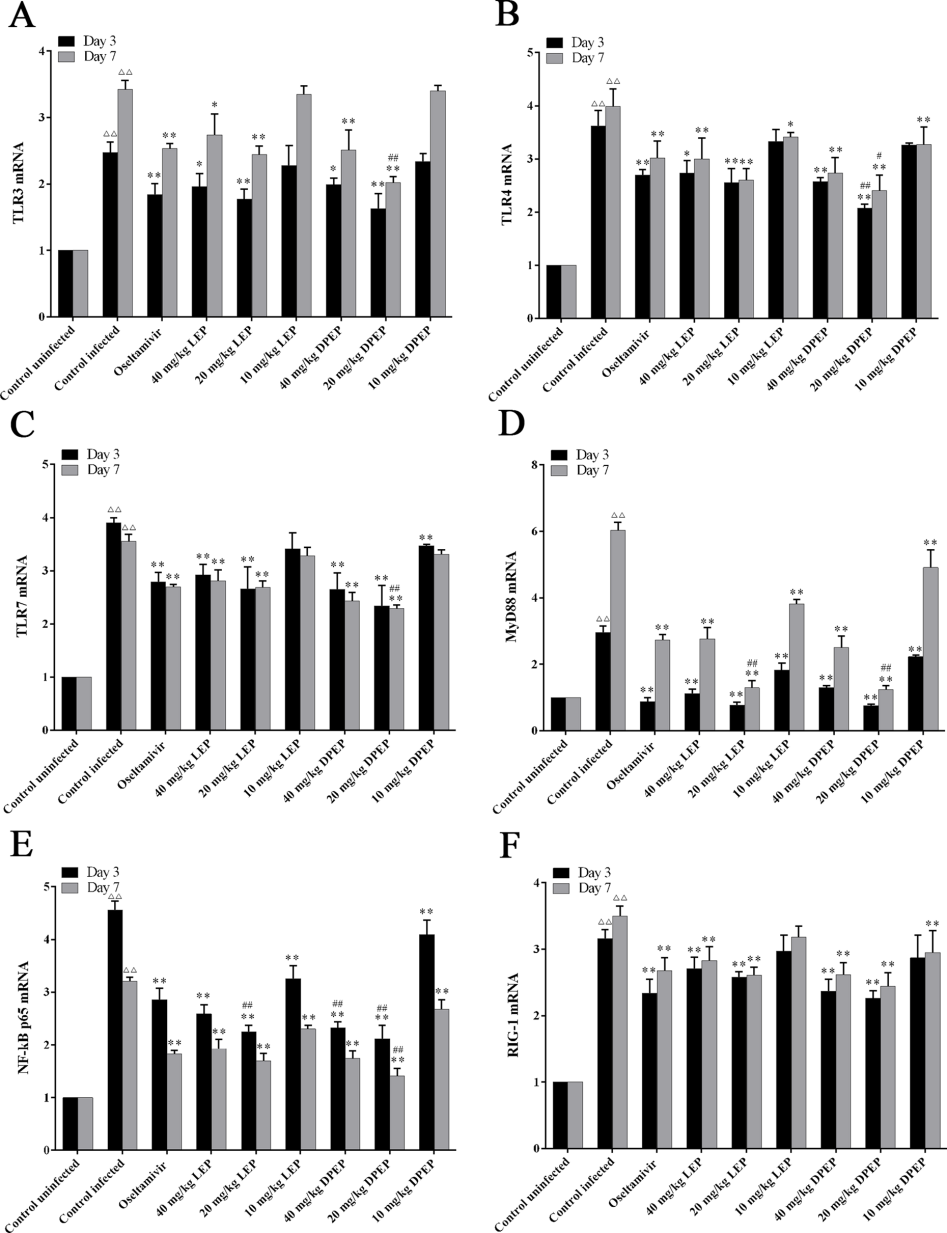
RT-PCR analysis of mRNA expression of genes related to the NF-κB signaling pathway. The key genes of the NF-κB signaling pathway include TLR3 **(A)**, TLR4 **(B)**, TLR7 **(C)**, MyD88 **(D)**, NF-κB p65 **(E)** and RIG-1 **(F)**. ^△△^ *P* < 0.01 compared with the control uninfected group; **P < 0.01 compared with the control infected group, *P < 0.05 compared with the control infected group; ^##^ *P* < 0.01 compared with oseltamivir group, ^#^ *P* < 0.05 compared with oseltamivir group.

To evaluate the effects of LEP and DPEP on the NF-κB p65 signaling pathway, mRNA expression levels of TLR3, TLR4, TLR7, MyD88, NF-κB p65 and RIG-1 in murine lung tissue were detected by RT-PCR. In comparison to the control uninfected group, mRNA expression levels of TLR3, TLR4, TLR7, MyD88, RIG-1 and NF-κB p65 in the control infected group were prominently increased on days 3 and 7 post-infection (all *P* < 0.01). At the 3rd and 7th days after infection, mRNA expression levels of genes mentioned above from the treatment groups with LEP and DPEP were obviously inhibited (*P* < 0.01 or *P* < 0.05), and more remarked down-regulation actions were especially observed in the LEP and DPEP 40 and 20 mg/kg groups (**Figure 11**).

### Effects of LEP and DPEP on Protein Expression of TLR4, TLR7, MyD88 and NF-κB p65 Genes

**Figure 12:**
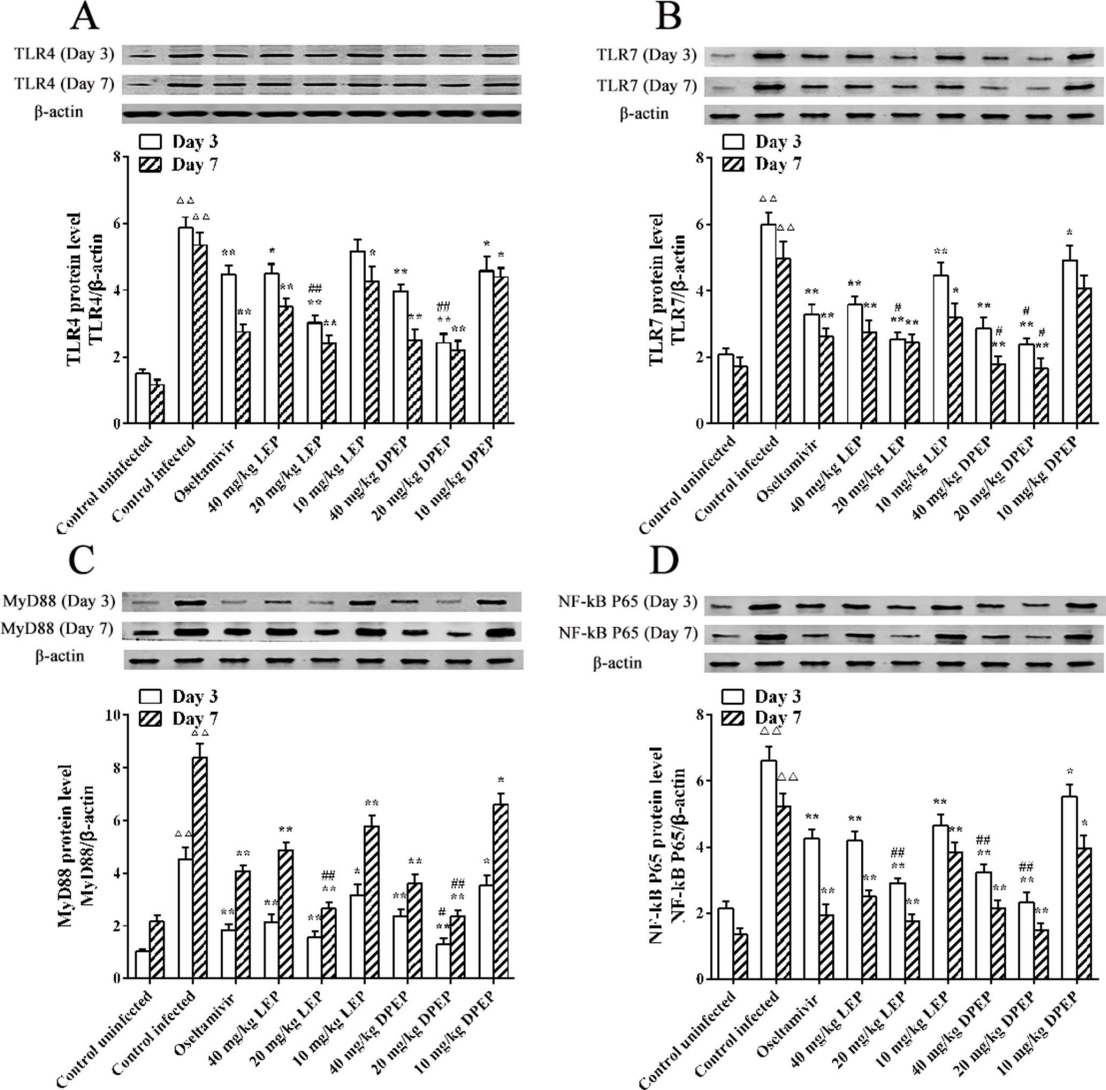
The protein expression TLR4 **(A)**, TLR7 **(B)**, MyD88 **(C)** and NF-κB p65 **(D)** in response to H1N1 stimulation. ^△△^ *P* < 0.01 compared with the control uninfected group; **P < 0.01 compared with the control infected group, *P < 0.05 compared with the control infected group; ^##^ *P* < 0.01 compared with the oseltamivir group, ^#^ *P* < 0.05 compared with the oseltamivir group.

Western blot experiment results showed that, at the 3rd and 7th days after infection, protein expression levels of TLR4, TLR7, MyD88 and NF-κB p65 in the murine lung tissues of the control infected group were significantly higher than those in the control uninfected group (all *P* < 0.01) (**Figure 12**). When treated with oseltamivir, LEP and DPEP, protein expression levels of the genes mentioned above were all markedly inhibited. Compared with the control infected group, protein expression levels of MyD88 and NF-κB p65 in the oseltamivir as well as the three doses of the LEP and DPEP groups were significantly down-regulated (*P* < 0.01 or *P* < 0.05). Decreased protein expression levels of TLR4 and TLR7 were found in the oseltamivir group as well as the LEP and DPEP 40 and 20 mg/kg groups (*P* < 0.01 or *P* < 0.05), while the inhibition effect in LEP or DPEP 10 mg/kg group was not obvious. Furthermore, in comparison to oseltamivir, 20 mg/kg LEP or DPEP could dramatically inhibit the protein expression of TLR4, TLR7 and NF-κB p65 on day 3 post-infection (*P* < 0.01 or *P* < 0.05).

## Discussion

The screening test for the effective components in MHT against influenza A virus showed that Ephedra alkaloids had significant protective effects on infected MDCK cells. After treatment with LMEP, LEP or DPEP, the A values in each experiment group significantly increased (all *P* < 0.01) in a dose-dependent manner, and the cell viability rate could reach 70% or higher at their respective TC_0_. As for the other six main ingredients in MHT, CMD and GA also could slightly increase the cell viability rate, but not obviously, and the remaining had little effect on the infected MDCK cells. These results demonstrated that the major antiviral components in MHT might be Ephedra alkaloids in Ephedra herb. Modern pharmacological studies indicate that the Ephedra herb has anti-inflammatory (Hiroshi et al., 1980), sudorific (Liu et al., 2006), antipyretic (Nagai et al., 2014), analgesic and anti-influenza effects (Mantani et al., 1999). Although Ephedra herb contains other constituents, such as phenolics, volatile oil and tannins, most pharmacological activities of the plant have been attributed to Ephedrine alkaloids (He et al., 2014; Li, 2015). Therefore, in the present study, based on the significant antiviral effects of LMEP, LEP and DPEP *in vitro*, we chose to conduct further mechanism studies on these three Ephedrine alkaloids.

It has been reported that LEP exerts a certain neuroprotective effect by inhibiting the apoptosis of hippocampal neurons cells (Xu et al., 2009); however, excessive usage of these alkaloids can be harmful to the human body, for example their corresponding neurotoxicity (Zheng et al., 2015b). It must strictly control the indications, contraindications and doses. To eliminate toxicity to MDCK cells due to overdose, the nontoxic concentrations of these three alkaloids were determined by MTT assay, and the results indicated that the cells’ survival rates were all higher than 90% when LMEP was at a concentration of 31.25 μg/ml and LEP or DPEP were at a concentration of 15.63 μg/ml. As a result, 31.25, 15.63 and 15.63 μg/ml were selected as the maximum doses of LMEP, LEP and DPEP in the subsequent experiments of this study, respectively. It is well known that the cycle of virus proliferation has six interconnected stages including adsorption, penetration, shelling, biosynthesis, assembling and release (Nagai et al., 2014). Hence, a time-of-drug-addition assay with one infectious cycle in MDCK cells was employed for investigating the antiviral efficiency and the action characteristics of LMEP, LEP and DPEP countering influenza A virus infection. The inhibitory effects of LMEP, LEP and DPEP on influenza virus replication were examined at different stages of replication using four different infection protocols. We found that three Ephedrine alkaloids all have obvious effects against influenza A virus by multiple ways. They stably and significantly prevented the viral infection by pre-treatment host cells prior to virus infection, limited treatment to 1 hour during virus infection, post-treatment host cells after virus infection and even pre-treatment of virus with drug. In addition, the ERs of four different infection protocols were different. When treated with the mean of post-treatment host cells after virus infection, the ERs of LMEP, LEP and DPEP were highest, being 64.16%, 71.57% and 63.52% at their highest concentrations, respectively. Furthermore, LMEP, LEP and DPEP exerted antiviral properties primarily by post-treatment host cells after virus infection. Therefore, the treatment way through post-treatment host cells after influenza A virus infection was chosen in the subsequent experiments of this study.

Our antiviral research data showed that LMEP, LEP and DPEP significantly decreased the virus load in MDCK cells infected by influenza A virus after 24 and 48 h administration (*P* < 0.01 or *P* < 0.05). Moreover, after 24 h treatment, the virus load in the LMEP 31.25 μg/ml, LEP 15.63 μg/ml and DPEP 15.63 μg/ml groups was significantly lower than that in oseltamivir group (*P* < 0.05), however, there was no difference between these three drug groups and the oseltamivir group after 48 h treatment. These results demonstrated that anti-influenza virus activities of LMEP, LEP and DPEP were more prominent at the early stage of the infection, and these Ephedra alkaloids were more helpful for the treatment of early phase influenza. This is consistent with the conclusion of our previous study that MHT is more effective for early phase influenza (Wei et al., 2018b).

The fatal consequence of influenza is eminently associated with a massive viral load and high cytokines imbalance, which causes a cytokine storm or hypercytokinemia (Xu et al., 2013). These cytokines include both pro- and anti-inflammatory cytokines (e.g. IL-, IL-6, IL-10, TNF-α and IFN type I & II) as well as the mononuclear chemoattractant chemokines (e.g. CXCL10, CXCL2) (Haghani et al., 2016). It has been reported that TNF-α level is directly related to the severity of histologic lung lesion or host cell apoptosis after infection (Aldridge et al., 2009). IL-6, regarding as a multifunctional cytokine, is necessary to control lung damage and avoid virus-induced death (Rong et al., 2016). It, along with TNF-α, can active and induce the differentiation of T and B lymphocytes and enhance the function of NK cells to kill target cells (Reeth et al., 2002; Liu, 2004). The innate immune system can promote innate anti-viral and anti-bacterial immunities, such as type I IFNs (Anderson, 2000). Type I IFNs, namely IFN-α and IFN-β, are regulated by IRF-3, IRF-7, NF-κB and several intracellular signaling molecules (Marie et al., 1998; Sato et al., 2000). To prevent the virus from replicating in the host, rapid production of IFN is necessary during viral infection (Choi et al., 2016). In the present study, we found that, compared with the control uninfected group, the influenza virus infection induced high levels of IFN-β, TNF-α and IL-6 secretion in MDCK cells of the control infected group after 24 and 48 h treatment, which further proved that influenza virus infection induced the body to produce a large number of inflammatory cytokines. When treated with LMEP, LEP and DPEP after 24 and 48 h, the levels of IFN-β and IL-6 were significantly increased, whereas TNF-α was significantly decreased in comparison to the control infected group. These results indicated that LMEP, LEP and DPEP could significantly reduce the content of TNF-α and inhibit the apoptosis of infected host cells, thereby reducing immune damage. Moreover, the level of IL-6 was increased by LMEP, LEP and DPEP, which suggested that these alkaloids might enhance the expression of IL-6 at the local inflammatory sites to activate the antiviral activity of NK cells or prevent the lesion from converting to chronic inflammation (Tian, 2013). As for IFN-β, we found that treatment with LMEP, LEP and DPEP increased not only its content in MDCK cell supernatant but also its mRNA expression in MDCK cells, showing that LEP, DPEP and LMEP inhibiting influenza A virus might be closely related to their induction effects on IFN-β high expression.

TLRs, the important pattern-recognition receptors in the innate immune system, have two major downstream signaling pathways through MyD88 and TIR domain- containing adapter-inducing IFN (TRIF), respectively (Martin and Wurfel, 2008; Downes and Marshall-Clarke, 2010). These signaling pathways all lead to the activation of NF-κB and expression of inflammatory mediators (He et al., 2015). Among TLRs, the main targets discriminated by TLR3 (Takeda, 2004; Li and Xiang, 2013) and TLR7 (Lai et al., 2016) are the double- and single-stranded RNA virus molecules, respectively, while TLR4 primarily recognizes bacterial lipopolysaccharide and viral envelope glycoproteins (An, 2013). When infected by a virus, intracellular TLRs recruit adapter proteins such as MyD88, TRAF3, TRIF, etc., to bind to the downstream kinase complex. By activating NF-κB and IRF3, the kinase complex further induces the expression of IFN-β and the corresponding inflammatory cytokines, to eventually exert the antiviral effect (Balachandran and Beg, 2011; Yin et al., 2016). Thus TLRs play an important role in the signal transduction pathways associated with defense of pathogenic microorganism such as influenza virus. In our study, mRNA expression levels of the relevant genes in the TLR3, TLR4 and TLR7 signaling pathways were investigated with RT-PCR assay. Our *in vitro* research results showed that mRNA expression of TLR3, TRAF3, IRF3, TLR4, TLR7, MyD88, TRAF6, NF-κB p65 and IFN-β in MDCK cells were up-regulated after being infected by influenza A virus, and there are marked differences between the control infected and uninfected groups. Treatment with three Ephedrine alkaloids significantly decreased mRNA expression of TLR3, TRAF3, IRF3, TLR4, TLR7, MyD88, TRAF6 and NF-κB p65 compared with the control infected group, showing that LMEP, LEP or DPEP played a role in anti-H1N1 influenza through multiple targets and pathways to regulate mRNA expression of related genes in the TLR signaling pathway. As shown in **Figure 6**, after 24 h and 48 h treatment, mRNA expression of TLR7, NF-κB p65, TLR3 and IRF3 in the three dose groups of LMEP, LEP and DPEP was significantly reduced (*P* < 0.01 or *P* < 0.05). Moreover, except for TRAF6, the medium and high doses of the three Ephedrine alkaloids all showed significant inhibitory action on mRNA expression of TLR3, TRAF3, IRF3, TLR4, TLR7, MyD88 and NF-κB p65, and that at 24 h was more noticeable than 48 h. These results further proved that LMEP, LEP and DPEP might be more appropriate to alleviate early stage influenza infection by regulating mRNA expression levels of the key genes in the TLR3, TLR4 and TLR7 signaling pathways.

Our cell test results confirmed that LMEP, LEP or DPEP had certain inhibitory effects on influenza A virus; in addition, LEP and DPEP had a more significant antiviral effect *in vitro*. Based on this, we further investigated the anti-influenza A virus effects and potential mechanisms of LEP and DPEP *in vivo*.

Studies have shown that excessive use of LEP and DPEP (≥50mg/kg) can produce some neuroexcitatory effects in mice (Jiang et al., 2004), but when the concentration of LEP or DPEP ranges from 40 mg/kg^-1^ to 80 mg/kg^-1^, there is no significant effect on the spontaneous activity of mice (Wang et al., 2001). Therefore, at the beginning of the animal experiment, we determined the reasonable dose by equivalent conversion of animal surface area, preliminary experimental screening of the research group, and consulting a large amount of literature, so as to ensure that the dose was within the safe range. The adult daily dose was converted into the high dose of mice, which was about 40 mg/kg.

The pneumonia occurring after infection with the influenza virus causes massive inflammatory cell infiltration, lung consolidation and weight gain in lung tissue, whereas the weight of infected mice often decreases, resulting in an increase in lung index (Peng et al., 2015). With the continuous replication and spread of the virus in the body, the viral load in the lung tissue continually increases. To monitor the severity of viral infection, lung index and the viral load in mouse lung tissue, as the important indicators, were determined in this study. Research has also reported that the main consequence of influenza virus infection is not the damage of respiratory epithelial cells directly mediated by the virus, but the immunopathological damage (Wiersma et al., 2014). In the present study, at days 3 and 7 post-infection, the viral load and lung indexes were significantly decreased, the lung damage was obviously relieved in the administration group, and the improvement effects were more significant on the 3rd day than on the 7th day after infection. These results indicate that LEP and DPEP have significant inhibitory effects on the *in vivo* replication of influenza virus, which causes the reduction of lung tissue lesions in mice after treatment. Additionally, it is suggested that LEP and DPEP may be more effective in inhibiting early influenza virus infection, which is consistent with the conclusion of our *in vitro* study.

Studies have shown that the thymus is the site of differentiation and development of T lymphocytes (Bi et al., 2010). Highly pathogenic influenza viruses are able to reach the thymus *via* dendritic cells and to interfere with T lymphocyte development, affecting the immune function (Vogel et al., 2010). Therefore, the thymus index reflects the immune status to some extent. In our study, 40 and 20 mg/kg LEP and DPEP significantly increased the thymus index of infected mice, indicating that LEP or DPEP has certain regulation effects on the immune function *in vivo*. However, influenza A virus had no significant influence on the spleen index of mice in this experiment. In viral pneumonia caused by the influenza virus, although the lymphocytes and macrophages in spleen are involved in the immune system, the spleen is not the target organ of the influenza virus, so the influenza virus infection has little effect on it (Bi et al., 2010).

Various cytokines play unique roles in the immune regulatory network of infection. Besides IL-6, TNF-α and IFN-β mentioned above, IL-1β can synthesize TNF-α by activating and stimulating endothelial cells and monocytes-macrophages, thus IL-1β is often used as the main target of therapeutic intervention in the treatment and pathogenesis of inflammatory diseases (Nan et al., 2011). IL-10, as an anti-inflammatory factor, whose key function is restriction of inflammatory response, can inhibit the secretion of pro-inflammatory cytokines and regulate the uncontrolled inflammatory reaction (Nan et al., 2016). IFN-γ, type II IFN is a critical cytokine for regulating both the innate and adaptive immune response, has antiviral and antiproliferative effects in the pathogenesis of influenza, and probably facilitates the induction of specific anti-influenza adaptive immunity (Sun and Metzger, 2008; Califano et al., 2018). As shown in **Figure 9** and **Figure 10**, compared with the control infected group, the IL-10 content in serum and the IFN-γ mRNA level in the lung tissue were significantly increased, whereas IL-1β content in serum and TNF-α mRNA level in the lung tissue were dramatically decreased when treated with LEP and DPEP. The results indicated that LEP and DPEP could improve the body’s innate immunity and regulate the synthesis and release of cytokines to work against influenza virus. As for IL-6, LEP and DPEP had little regulation effect on its secretion in serum, and this is consistent with the conclusion of our *in vitro* study.

As we discussed above, in addition to TLRs, retinoic acid-inducible gene-1 (RIG-1) is another major pattern-recognition receptor associated with viruses and is found in cytoplasmic matrices (Lee and Yen, 2012). TLR4 and TLR7 mainly depend on MyD88 and ultimately activate NF-κB p65, then induce type I interferon, inflammation response, etc., to exert antiviral action, while TLR3 and RIG-1 are non-MyD88-dependent, can regulate adapter protein TRIF to activate IRF3 or NF-κB p65, finally induce type I interferon or inflammatory cytokines, etc. to play the antiviral activity (Yoneyama and Fujita, 2007). In this work, mRNA levels of TLR3, TLR4, TLR7, MyD88, NF-κB p65 and RIG-1 in the murine lung tissue from the control infected group were significantly increased compared with the control uninfected group at days 3 and 7 post-infection, suggesting influenza virus activated the TLR3, TLR4, TLR7 and RIG-1 signaling pathways after infection. Nevertheless, LEP and DPEP markedly inhibited mRNA levels of genes mentioned above, indicating that LEP and DPEP had protective effects on infected mice by regulating the mRNA expression of TLR3, TLR4, TLR7, MyD88, NF-κB p65 and RIG-1 to lighten inflammation and improve immune function with the development of influenza virus-induced pneumonia. Additionally, the results of western blot test further demonstrated that LEP and DPEP significantly inhibited the high protein expression of TLR4, TLR7, MyD88 and NF-κB p65 caused by influenza A virus infection, and the inhibitory effect was more obvious on the 3rd day of infection. This study revealed that LEP and DPEP could down-regulate the activity of NF-κB p65 by regulating MyD88-dependent signaling pathways of TLR4 and TLR7, thereby exerting their antiviral effects, and the treatment effects might be more significant in the early phase of infection.

## Conclusions

In summary, our results demonstrate that, on the premise of nontoxicity to host cells, LMEP, LEP and DPEP in MHT have obvious anti-influenza A virus effects *in vitro* by way of pre-treatment of host cells prior to virus infection, limited treatment to 1 hour during virus infection, post-treatment host cells after virus infection and even pre-treatment of virus with drug, which may be closely related to the inhibition of viral replication and the modulation of inflammatory response by regulating the production of inflammation cytokines and mRNA expression levels of relevant genes in the TLR3, TLR4 and TLR7 signaling pathways. Furthermore, LEP and DPEP have certain protective effects on the influenza virus-infected mice, which may be associated with their abilities of effectively alleviating lung injury, improving the immunologic function of infected mice by regulating the imbalance of inflammatory cytokine secretion and adjusting the mRNA expression levels of TLR3 and RIG-1 as well as the mRNA and protein expression levels of TLR4, TLR7, MyD88 and NF-κB p65. These findings show Ephedra alkaloids may have potential utility in the clinical management.

Although Ephedra alkaloids have significant antiviral effect *in vivo* and *in vitro*, they also have neurotoxicity. It is amazing that MHT, as a classical prescription of traditional Chinese medicine, has been used in the treatment of influenza and asthma for thousands of years, which indicates the safety of MHT. Considering the reported toxicity of Ephedra alkaloids and the safety of MHT in clinical use, MHT should be the better choice for treatment of influenza between the administration of MHT and its active components. The overall results of this experiment demonstrate that Ephedra alkaloids may be the main antiviral components in MHT. We speculate that the other three drugs in MHT may promote the exertion of the pharmacodynamics of Ephedra, and the other components in MHT may have a good inhibitory effect on the toxicity of Ephedra alkaloids. The purpose of this study is to screen what exactly are the antiviral components in MHT. Therefore, we will further investigate the synergistic effect and compatibility mechanism of increasing effectiveness and reducing toxicity of other components in MHT combined with Ephedra alkaloids in our subsequent studies. Moreover, LEP and DPEP may be used as adjuvants at lower concentration, and in this respect, the investigation of possible synergistic effects with LEP, DPEP and oseltamivir are worth performing as well in the future.

## Data Availability

The raw data supporting the conclusions of this manuscript are available upon reasonable request to the corresponding author.

## Ethics Statement

This study was carried out in accordance with the recommendations of the Guide for the Care and Use of Laboratory Animals (NIH Publications, No.80-23, revised in 1996). The protocol was approved by Animal Ethics Committee of Zhejiang Chinese Medical University.

## Author Contributions

WW, HZ, LY, and HD performed the experiments and data analysis. WW, YH, and CS wrote the manuscript. YH and HW conceived and designed the study. Yi L supervised the whole experimental work. HD and YH revised the manuscript.

## Funding

This work was financially supported by the National Natural Science Foundation of China (Nos. 81573868 and 81603531), Zhejiang Provincial Natural Science Foundation of China (No. LZ18H270001), and Zhejiang Provincial Program for the Cultivation of High-level Innovative Health talents.

## Conflict of Interest Statement

The authors declare that the research was conducted in the absence of any commercial or financial relationships that could be construed as a potential conflict of interest.
