# Supplementary material for: Screening of Antiviral Components of Ma Huang Tang and Investigation on the Ephedra Alkaloids Efficacy on Influenza Virus Type A
Source: Front Pharmacol. 2019 Sep 10;10:961. doi: 10.3389/fphar.2019.00961 (PMC6747051; doi:10.3389/fphar.2019.00961)
Supplement: Supplementary file 1 [file Table_1.docx]

**Table S1** The primers sequences and product fragments of genes for RT-PCR analysis

| Gene | Primer sequence (5’-3’) | Length of primer/bp |
| --- | --- | --- |
| GAPDH | F: GTGACACCCACTCTTCCACC | 162 |
|  | R: GTGGTCCAGGAGGCTCTTAC |  |
| M1 | F: TCATTGGGATCTTGCACTTG | 117 |
|  | R: ACTTTGGCACTCCTTCCGTA |  |
| TLR4 | F: AACTGTATCGCCTCCTCAGC | 136 |
|  | R: GCATCCTCTGTTCCTTCTGG |  |
| TLR7 | F: GGTGAAGTTGGCTTCTGCTC | 109 |
|  | R: TGCAACTCCTTGCATACTCG |  |
| MyD88 | F: CCGGATGGTAGTGGTTGTCT | 91 |
|  | R: GGCACCTGGAGAGAGACTGA |  |
| TRAF6 | F: TTGTCTCCAGTGCCAACGTA | 107 |
|  | R: CCATCAATGCAGCACAGTTC |  |
| TLR3 | F: GCAACACCCAGCTACACAGA | 131 |
|  | R: ATGTGGAAGCCAGACAAAGG |  |
| TRAF3 | F: AGCAGACAGCATGAAGAGCA | 111 |
|  | R: GACTCCAGCAAGCCTGTGTT |  |
| IRF3 | F: ACCAAGAAGCTGGTGATGCT | 107 |
|  | R: TGCAGGTCCACAGTGTTCTC |  |
| NF-κB p65 | F: CAGCCATGGACGACCTGTTTC | 113 |
|  | R: CTGCTTGGGTTGCTCGATGA |  |
| IFN-β | F: CCAGTTCCAGAAGGAGGACA | 120 |
|  | R: TGTCCCAGGTGAAGTTTTCC |  |

**Table S2** The primer sequences and product fragments of genes for RT-PCR analysis

| Gene | Primer sequence (5’-3’) | Length of primer/bp |
| --- | --- | --- |
| M1 | F: TCATTGGGATCTTGCACTTG | 117 |
|  | R: ACTTTGGCACTCCTTCCGTA |  |
| GAPDH | F: GCAAGTTCAACGGCACAG | 141 |
|  | R: CGCCAGTAGACTCCACGAC |  |
| TLR3 | F: AAAGGGTGTTCCTCTTATC | 212 |
|  | R: AAGTTGGTAGGTGGTAATC |  |
| TLR4 | F: GCACTGACACCTTCCTTTCC | 101 |
|  | R: GCCTTAGCCTCTTCTCCTTCA |  |
| TLR7 | F: CTTGACCTAAGTGGAAATTG | 154 |
|  | R: CATGCTGAAGAGAATTACTG |  |
| MyD88 | F: ACTCGCAGTTTGTTGGATG | 183 |
|  | R: CACCTGTAAAGGCTTCTCG |  |
| NF-κB p65 | F: TCAATGGCTACACAGGACCA | 185 |
|  | R: TCGCTTCTTCACACACTGGA |  |
| RIG-1 | F: GCGTTGGAGATGCTAAGACC | 121 |
|  | R: ACGACCTGTTCCAGTTCTGC |  |
| TNF-α | F: CCAAAGGGATGAGAAGTTCC | 133 |
|  | R: CTCCACTTGGTGGTTTGCTA |  |
| IFN-γ | F: AGGCCATCAGCAACAACATA | 150 |
|  | R: TGAGCTCATTGAATGCTTGG |  |

**Table S3** TC_50_ and TC_0_ of drugs for MDCK cells

| Drugs | TC_50_ (μg/ml) | TC_0_ (μg/ml) |
| --- | --- | --- |
| LMEP | 216.77 | 31.25 |
| LEP | 215.28 | 15.63 |
| DPEP | 237.14 | 15.63 |
| CAO | 390.84 | 125.00 |
| CMA | 610.94 | 62.50 |
| CMD | 75.86 | 15.63 |
| LIQ | 680.77 | 250.00 |
| GA | 663.74 | 125.00 |
| AMY | 563.64 | 31.25 |
| Oseltamivir | 500.00 | 100.00 |

**Table S4** Effect of LMEP against influenza A virus *in vitro* (*$\bar{x}$± s, n=6*)

| Group | Concentration (μg/ml) | A | Cell survival rate (%) |
| --- | --- | --- | --- |
| LMEP | 0.98 | 0.59±0.01^**^ | 57.33 |
|  | 1.95 | 0.65±0.02^**^ | 63.17 |
|  | 3.91 | 0.69±0.01^**^ | 66.57 |
|  | 7.81 | 0.72±0.02^**^ | 70.26 |
|  | 15.63 | 0.77±0.01^**^ | 75.08 |
|  | 31.25 | 0.80±0.03^**^ | 77.75 |
| Oseltamivir | 10.00 | 0.85±0.01^**^ | 82.70 |
| Control uninfected | / | 1.03±0.05 | 100 |
| Control infected | / | 0.38±0.04^△△^ | 36.89 |

Notes: “A value” represents absorbance. ^△△^*P <* 0.01 compared with the control uninfected group; ^**^*P <* 0.01 compared with the control infected group, ^*^*P <* 0.05 compared with the control infected group; ^##^*P <* 0.01 compared with oseltamivir group, ^#^*P <* 0.05 compared with oseltamivir group.

**Table S5** Effect of LEP against influenza A virus *in vitro* (*$\bar{x}$± s, n=6*)

| Group | Concentration (μg/ml) | A | Cell survival rate (%) |
| --- | --- | --- | --- |
| LEP | 0.49 | 0.43±0.01^**^ | 42.57 |
|  | 0.98 | 0.52±0.01^**^ | 51.58 |
|  | 1.95 | 0.58±0.02^**^ | 57.82 |
|  | 3.91 | 0.62±0.01^**^ | 61.47 |
|  | 7.81 | 0.68±0.02^**^ | 67.23 |
|  | 15.63 | 0.75±0.01^**^ | 74.55 |
| Oseltamivir | 10.00 | 0.84±0.02^**^ | 83.47 |
| Control uninfected | / | 1.01±0.05 | 100 |
| Control infected | / | 0.29±0.04^△△^ | 29.11 |

Notes: “A value” represents absorbance. ^△△^*P <* 0.01 compared with the control uninfected group; ^**^*P <* 0.01 compared with the control infected group, ^*^*P <* 0.05 compared with the control infected group; ^##^*P <* 0.01 compared with oseltamivir group, ^#^*P <* 0.05 compared with oseltamivir group.

**Table S6** Effect of DPEP against influenza A virus *in vitro* (*$\bar{x}$± s, n=6*)

| Group | Concentration (μg/ml) | A | Cell survival rate (%) |
| --- | --- | --- | --- |
| DPEP | 0.49 | 0.55±0.03^**^ | 45.26 |
|  | 0.98 | 0.67±0.02^**^ | 54.66 |
|  | 1.95 | 0.75±0.01^**^ | 61.19 |
|  | 3.91 | 0.78±0.03^**^ | 63.89 |
|  | 7.81 | 0.89±0.01^**^ | 72.47 |
|  | 15.63 | 0.99±0.02^**^ | 80.72 |
| Oseltamivir | 10.00 | 1.01±0.01^**^ | 82.27 |
| Control uninfected | / | 1.22±0.05 | 100 |
| Control infected | / | 0.39±0.03^△△^ | 32.19 |

Notes: “A value” represents absorbance.^△△^*P <* 0.01 compared with the control uninfected group; ^**^*P <* 0.01 compared with the control infected group, ^*^*P <* 0.05 compared with the control infected group; ^##^*P <* 0.01 compared with oseltamivir group, ^#^*P <* 0.05 compared with oseltamivir group.

**Table S7** Effect of CMD against influenza A virus *in vitro* (*$\bar{x}$± s, n=6*)

| Group | Concentration (μg/ml) | A | Cell survival rate (%) |
| --- | --- | --- | --- |
| CMD | 0.49 | 0.32±0.03 | 27.04 |
|  | 0.98 | 0.33±0.03 | 27.64 |
|  | 1.95 | 0.37±0.01^*^ | 31.21 |
|  | 3.91 | 0.41±0.02^**^ | 34.44 |
|  | 7.81 | 0.50±0.02^**^ | 42.52 |
|  | 15.63 | 0.58±0.04^**^ | 49.57 |
| Oseltamivir | 10.00 | 0.96±0.07^**^ | 81.97 |
| Control uninfected | / | 1.18±0.07 | 100 |
| Control infected | / | 0.30±0.04^△△^ | 25.34 |

Notes: “A value” represents absorbance. ^△△^*P <* 0.01 compared with the control uninfected group; ^**^*P <* 0.01 compared with the control infected group, ^*^*P <* 0.05 compared with the control infected group; ^##^*P <* 0.01 compared with oseltamivir group, ^#^*P <* 0.05 compared with oseltamivir group.

**Table S8** Effect of GA against influenza A virus *in vitro* (*$\bar{x}$± s, n=6*)

| Group | Concentration (μg/ml) | A | Cell survival rate (%) |
| --- | --- | --- | --- |
| GA | 3.91 | 0.35±0.03 | 31.08 |
|  | 7.81 | 0.37±0.02^*^ | 32.77 |
|  | 15.63 | 0.39±0.03^**^ | 34.37 |
|  | 31.25 | 0.48±0.05^**^ | 43.01 |
|  | 62.50 | 0.52±0.04^**^ | 45.95 |
|  | 125 | 0.53±0.04^**^ | 47.20 |
| Oseltamivir | 10.00 | 0.91±0.06^**^ | 80.68 |
| Control uninfected | / | 1.12±0.07 | 100 |
| Control infected | / | 0.31±0.03^△△^ | 27.87 |

Notes: “A value” represents absorbance. ^△△^*P <* 0.01 compared with the control uninfected group; ^**^*P <* 0.01 compared with the control infected group, ^*^*P <* 0.05 compared with the control infected group; ^##^*P <* 0.01 compared with oseltamivir group, ^#^*P <* 0.05 compared with oseltamivir group.

**Table S9** Effect of CAO against influenza A virus *in vitro* (*$\bar{x}$± s, n=6*)

| Group | Concentration (μg/ml) | A | Cell survival rate (%) |
| --- | --- | --- | --- |
| CAO | 3.91 | 0.32±0.03 | 25.02 |
|  | 7.81 | 0.33±0.05 | 25.89 |
|  | 15.63 | 0.34±0.02 | 26.69 |
|  | 31.25 | 0.33±0.02 | 26.45 |
|  | 62.5 | 0.38±0.05^*^ | 29.94 |
|  | 125 | 0.41±0.05^**^ | 32.57 |
| Oseltamivir | 10.00 | 1.03±0.01^**^ | 81.81 |
| Control uninfected | / | 1.26±0.05 | 100 |
| Control infected | / | 0.31±0.02^△△^ | 24.31 |

Notes: “A value” represents absorbance. ^△△^*P <* 0.01 compared with the control uninfected group; ^**^*P <* 0.01 compared with the control infected group, ^*^*P <* 0.05 compared with the control infected group; ^##^*P <* 0.01 compared with oseltamivir group, ^#^*P <* 0.05 compared with oseltamivir group.

**Table S10** Effect of CMA against influenza A virus *in vitro* (*$\bar{x}$± s, n=6*)

| Group | Concentration (μg/ml) | A | Cell survival rate (%) |
| --- | --- | --- | --- |
| CMA | 1.95 | 0.31±0.03 | 27.73 |
|  | 3.91 | 0.31±0.01 | 27.91 |
|  | 7.81 | 0.35±0.04 | 30.95 |
|  | 15.63 | 0.39±0.04^**^ | 34.79 |
|  | 31.25 | 0.38±0.04^*^ | 33.54 |
|  | 62.50 | 0.43±0.07^*^ | 38.19 |
| Oseltamivir | 10.00 | 0.89±0.03^**^ | 79.87 |
| Control uninfected | / | 1.12±0.06 | 100 |
| Control infected | / | 0.31±0.03^△△^ | 27.64 |

Notes: “A value” represents absorbance. ^△△^*P <* 0.01 compared with the control uninfected group; ^**^*P <* 0.01 compared with the control infected group, ^*^*P <* 0.05 compared with the control infected group; ^##^*P <* 0.01 compared with oseltamivir group, ^#^*P <* 0.05 compared with oseltamivir group.

**Table S11** Effect of LIQ against influenza A virus *in vitro* (*$\bar{x}$± s, n=6*)

| Group | Concentration (μg/ml) | A | Cell survival rate (%) |
| --- | --- | --- | --- |
| LIQ | 7.81 | 0.31±0.02 | 26.62 |
|  | 15.63 | 0.32±0.03 | 26.88 |
|  | 31.25 | 0.33±0.03 | 28.41 |
|  | 62.50 | 0.33±0.01 | 27.99 |
|  | 125 | 0.38±0.02^**^ | 32.25 |
|  | 250 | 0.40±0.03^**^ | 33.70 |
| Oseltamivir | 10.00 | 0.94±0.07^**^ | 80.03 |
| Control uninfected | / | 1.17±0.11 | 100 |
| Control infected | / | 0.31±0.03^△△^ | 26.28 |

Notes: “A value” represents absorbance. ^△△^*P <* 0.01 compared with the control uninfected group; ^**^*P <* 0.01 compared with the control infected group, ^*^*P <* 0.05 compared with the control infected group; ^##^*P <* 0.01 compared with oseltamivir group, ^#^*P <* 0.05 compared with oseltamivir group.

**Table S12** Effect of AMY against influenza A virus in vitro (*$\bar{x}$± s, n=6*)

| Group | Concentration (μg/ml) | | A | Cell survival rate (%) |
| --- | --- | --- | --- | --- |
| AMY | | 0.98 | 0.33±0.03 | 26.23 |
|  | 1.95 | | 0.34±0.06 | 27.03 |
|  | 3.91 | | 0.36±0.04^*^ | 29.04 |
|  | 7.81 | | 0.35±0.02^*^ | 27.92 |
|  | 15.63 | | 0.35±0.03^*^ | 28.24 |
|  | 31.25 | | 0.36±0.02^*^ | 28.88 |
| Oseltamivir | 10.00 | | 0.98±0.01^**^ | 78.52 |
| Control uninfected | / | | 1.24±0.13 | 100 |
| Control infected | / | | 0.31±0.02^△△^ | 24.78 |

Notes: “A value” represents absorbance. ^△△^*P <* 0.01 compared with the control uninfected group; ^**^*P <* 0.01 compared with the control infected group, ^*^*P <* 0.05 compared with the control infected group; ^##^*P <* 0.01 compared with oseltamivir group, ^#^*P <* 0.05 compared with oseltamivir group.
